# Potential Causal Relationship Between Hypertension and Type 2 Diabetic Nephropathy: Integrating Mendelian Randomization Evidence with Global Burden of Disease 2021 Analysis

**DOI:** 10.3390/healthcare14121725

**Published:** 2026-06-15

**Authors:** Dongsen Hu, Runze Wang, Pengfei Xie, Yexin Chen, Lili Zhang, Linhua Zhao

**Affiliations:** 1 Guang’anmen Hospital, China Academy of Chinese Medical Sciences, Beijing 100053, China; bjbucmhds@126.com (D.H.); xie13071021023@163.com (P.X.); 2 Dongzhimen Hospital, Beijing University of Chinese Medicine, Beijing 100700, China; 18811203805@163.com (R.W.); nbchenyexin@163.com (Y.C.)

**Keywords:** hypertension, diabetic nephropathy, Mendelian randomization, Global Burden of Disease, type 2 diabetic nephropathy attributable to hypertension

## Abstract

**Highlights:**

**What are the main findings?**

- Genetically predicted hypertension was associated with a higher risk of diabetic nephropathy in the Mendelian randomization analyses.
- Global deaths and DALYs of hypertension-attributable type 2 diabetic nephropathy increased from 1990 to 2021 and are projected to continue rising through 2045.

**What are the implications of the main findings?**

- Blood pressure control should be prioritized in patients with type 2 diabetes to reduce kidney-related complications.
- Prevention and screening strategies should focus on women, older adults, and populations in lower-SDI regions with the highest burden.

**Abstract:**

**Background:** Hypertension (HTN) and type 2 diabetes mellitus are major global health challenges, and diabetic nephropathy (DN) is a critical complication of diabetes. Although observational studies link HTN to DN progression, causal evidence remains limited. We investigated the potential causal relationship between HTN and DN and quantified the global burden of HTN-attributable type 2 diabetic nephropathy (HTN-T2DN). **Methods:** We integrated two-sample Mendelian randomization (MR), Bayesian weighted MR, and sensitivity analyses with Global Burden of Disease (GBD) 2021 analyses. The burden of HTN-T2DN was assessed from 1990 to 2021 and projected to 2045. **Results:** MR provided genetic evidence supporting a potential causal role of HTN in DN (inverse-variance weighted odds ratio = 4.219, 95% CI: 1.807–9.853; *p* = 0.001). Globally, HTN-T2DN deaths increased to 50,689 and DALYs to 1,151,216 in 2021. Females had higher age-standardized mortality and DALY rates than males, and low-middle sociodemographic index (SDI) regions had the highest burden. By 2045, deaths and DALYs were projected to reach 162,392 and 4.04 million, respectively. **Conclusions:** HTN may play a potential causal role in DN development and progression. Strengthened blood pressure control, early screening, and tailored policies are essential, particularly for women, older adults, and populations in lower-SDI settings.

## 1. Introduction

Hypertension (HTN) and type 2 diabetes mellitus (T2DM) rank as two of the most prevalent non-communicable diseases globally, contributing to significant public health burdens [1,2]. Diabetic nephropathy (DN), a critical microvascular complication of T2DM, represents the primary pathway to end-stage renal disease (ESRD) [3]. Epidemiological surveillance data reveal a persistent upward trajectory in the global disease burden of DN, with projection models suggesting accelerated progression without targeted therapeutic strategies [4]. The frequent comorbidity of HTN and DN in T2DM patients establishes a high-risk clinical phenotype.

The synergistic public health impact of the concurrent burden of HTN and type 2 diabetic nephropathy (T2DN) is well documented across epidemiological studies. HTN is a strong predictor of CKD in patients with T2DM, and the risk increases further with the duration of diabetes [5]. In a retrospective cohort of 914 T2DM patients, systolic blood pressure (SBP) demonstrated a positive gradient association with chronic kidney disease (CKD) risk [6]. Higher CKD prevalence was observed in T2DM patients with comorbid HTN [6]. A 10-year prospective cohort study (*n* = 1682) demonstrated that HTN was significantly associated with accelerated glomerular filtration rate (eGFR) decline in T2DM patients [7]. A nationwide Thai cohort analysis (*n* = 595 hospitals) confirmed that HbA1c ≤ 7% is associated with slowed CKD progression, while HTN remained an independent risk factor for CKD progression after multivariable adjustment. This indicates that optimal nephroprotection requires coordinated management of both hyperglycemia and HTN [8].

While extensive observational evidence has characterized associations between HTN and T2DN, conventional epidemiological studies cannot establish causal directionality. Confounding factors such as obesity, smoking, alcohol use, and socioeconomic status make it difficult to disentangle the causal relationship. The independent causal role of HTN in the development of DN remains to be established. Mendelian randomization (MR) uses genetic variants strongly linked to target exposures as instrumental variables (IVs) to assess causal effects on outcomes. By leveraging alleles established at conception (largely protected from confounding and reverse causation), MR strengthens the validity of causal inference [9].

Bidirectional MR confirmed mutual causation, demonstrating that genetically elevated albuminuria increased blood pressure while elevated blood pressure exacerbated albuminuria [10]. In addition, previous work has integrated MR and GBD analyses to explore the causal relationship between obesity and T2DN [11]. In the present study, we integrated MR and GBD analyses to genetically assess the causal role of HTN in DN and to quantify the global burden of HTN-attributable T2DN (HTN-T2DN) from 1990 to 2045. This dual approach provides genetically informed causal evidence together with policy-relevant estimates and forecasts to guide future public health interventions.

## 2. Materials and Methods

### 2.1. Study Design

This study was conducted in two phases. The first phase involved causal relationship analyses. Two-sample MR and Bayesian weighted MR (BWMR) were employed to evaluate the causal relationship between HTN and DN while minimizing confounding and reverse causation. The second phase focused on disease burden analysis. Based on GBD 2021 data, we analyzed the spatiotemporal characteristics of HTN-T2DN epidemiology from 1990 to 2021.

This study was reported in accordance with STROBE-MR (STROBE-MR checklist) [12]. The study protocol was not preregistered.

### 2.2. Data Sources

GWAS data for HTN and DN were used in this MR study and were sourced from the IEU OpenGWAS (https://gwas.mrcieu.ac.uk/) (accessed on 8 June 2025) and the GWAS Catalog (https://www.ebi.ac.uk/gwas/) (accessed on 8 June 2025) [13,14]. Detailed information is presented in Table 1. To minimize the effects of population stratification and temporal bias, we restricted the datasets to consistent ethnicity and data collection periods, thereby enhancing the robustness of causal inference.

Data on the global burden of HTN-T2DN were obtained from the GBD 2021 dataset (https://vizhub.healthdata.org/gbd-results/) (accessed on 8 June 2025) curated by the Institute for Health Metrics and Evaluation (IHME). This comprehensive dataset systematically evaluates disease burden across 204 countries and territories, encompassing 371 diseases and 88 risk factors. In this study, HTN-T2DN was defined as the burden of CKD due to T2DM attributable to the metabolic risk factor of high SBP. We extracted annual mortality and disability-adjusted life years (DALYs) from 1990 to 2021, including counts, rates, and age-standardized rates. Data were stratified by sex, age, location, and sociodemographic index (SDI) [15]. Estimates were derived using the GBD Comparative Risk Assessment (CRA) framework. Instead of a simple summation, the attributable burden was calculated as the product of the total outcome burden and the Population Attributable Fraction (PAF). The PAF integrates exposure distributions, relative risk functions, and the Theoretical Minimum Risk Exposure Level (TMREL), with mediation adjustments applied to prevent double-counting of combined risks [16,17,18].

### 2.3. Ethical Considerations

All original studies were approved by their respective institutional ethics review boards. Because all analyses in this study were based on publicly available, de-identified summary-level data, no additional ethical approval or informed consent was required for the present analysis.

### 2.4. MR Analysis

#### 2.4.1. IV Screening

**Figure 1:**
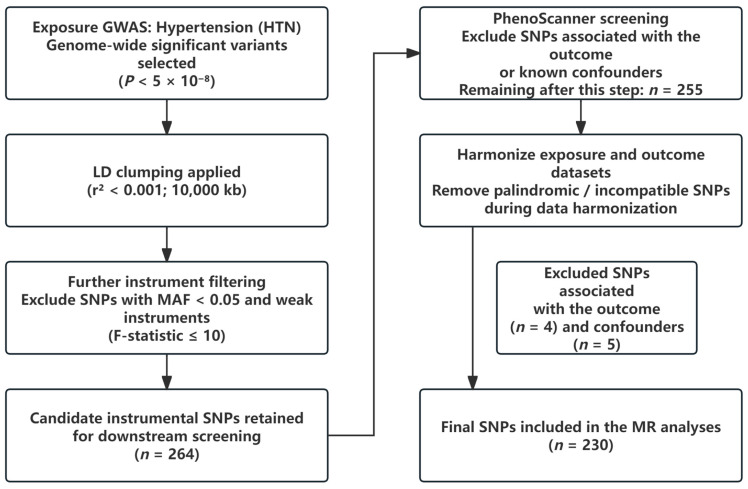
Flow diagram of SNP selection and exclusion for the MR analysis. SNP, single nucleotide polymorphism; MR, Mendelian randomization; GWAS, genome-wide association study; LD, linkage disequilibrium; MAF, minor allele frequency.

MR analysis is based on three key assumptions: (1) the IVs are directly associated with the exposure; (2) the IVs are independent of potential confounders; (3) the IVs influence the outcome only through the exposure [19]. SNPs significantly associated with the exposure traits were selected using a genome-wide significance threshold of *p* < 5.0 × 10^−8^. To eliminate linkage disequilibrium, an r^2^ threshold of 0.001 and a clumping window size of 10,000 kb were applied. SNPs with a minor allele frequency (MAF) < 0.05 were excluded. The strength of the IVs was assessed using the F-statistic, calculated as F = β^2^/SE^2^, where β represents the allele effect size and SE is the standard error. An F-statistic greater than 10 indicates the absence of weak instrument bias [20]. SNPs associated with the outcome or potential confounders were excluded. During harmonization of the exposure and outcome datasets, we set the parameter ‘action = 3’ and excluded palindromic SNPs (Figure 1).

#### 2.4.2. Statistical Analysis of MR

We used five models, including MR-Egger regression, weighted mode (WM), weighted median (WME), simple mode (SM), and inverse-variance weighted (IVW), to evaluate the potential causal relationship between HTN (exposure) and DN (outcome). These methods can evaluate the robustness and reliability of causal estimates under different assumptions, with IVW used as the primary analytical method for assessing causality [21]. We also employed the Steiger test to examine the directionality of causal relationships. To enhance the robustness of causal inference, we further employed BWMR to verify the existence of a causal relationship between HTN and DN. This model automatically adjusts for potential horizontal pleiotropy of IVs through a Bayesian hierarchical structure and adopts a weighting strategy to reduce interference from heterogeneity [22].

#### 2.4.3. Sensitivity Analysis

To evaluate and adjust for potential pleiotropy and heterogeneity in the causal estimates, we performed several sensitivity analyses. Cochran’s Q test was used to assess heterogeneity in the individual effect estimates generated by each genetic variant. A *p*-value < 0.05 was considered indicative of heterogeneity, in which case results from the inverse-variance weighted random-effects model were emphasized [23]. MR-Egger regression was used to assess directional pleiotropy. An intercept close to zero suggests a lower likelihood of genetic pleiotropy, and a *p*-value > 0.05 indicates that horizontal pleiotropy is unlikely to bias the causal estimate [24]. We also performed leave-one-out analyses to identify influential single nucleotide polymorphisms, and we generated scatter plots and funnel plots to assess robustness. MR-PRESSO was applied to evaluate the presence of outlier or pleiotropic variants [25,26].

All MR analyses were performed using R software (version 4.4.1) with the TwoSampleMR package.

### 2.5. GBD Analysis

#### 2.5.1. Statistical Analysis of GBD

This study incorporated two primary indicators: absolute case numbers and age-standardized rates (ASR) [27]. Absolute numbers represented crude case counts, while ASR was calculated by weighting age-specific rates (stratified in 5-year intervals) against a standardized population structure, with results expressed per 100,000 population. All estimates were reported with 95% uncertainty intervals (UI), computed through aggregation and weighting algorithms employed in the GBD 2021 methodology [28]. To investigate associations between disease burden and socioeconomic development, SDI data from 1990 to 2021 were integrated. Countries were categorized into five SDI groups based on economic development levels: low (<0.46), low-middle (0.46 to 0.60), middle (0.61 to 0.69), high-middle (0.70 to 0.81), and high (>0.81).

#### 2.5.2. Trend Analysis

This study employed Joinpoint regression analysis (Joinpoint Regression Program version 5.0) implemented using R software to model temporal trends in disease burden from 1990 to 2021. The model identified significant inflection points and estimated annual percentage change (APC) for each temporal segment. Overall trends were summarized by calculating the average annual percentage change (AAPC) and its 95% confidence interval (CI) [29,30]. A *p*-value < 0.05 was regarded as statistically significant.

#### 2.5.3. Predictive Analysis

To project future disease burden, a Bayesian age-period-cohort (BAPC) model was used to forecast mortality, DALYs, age-standardized mortality rate (ASMR), and age-standardized DALY rate (ASDR) from 2022 to 2045. By integrating historical trends, age structure, and population dynamics through the integrated nested Laplace approximation (INLA) algorithm, the model generated projections with 95% UI.

#### 2.5.4. Frontier Analysis

To explore country-level performance in controlling HTN-T2DN burden, we conducted frontier analyses. Countries were categorized according to SDI, and the frontier represented the minimum achievable burden for a given level of development. Countries farther from the frontier were interpreted as having a larger gap between observed and potentially achievable burden reduction. We highlighted countries closest to the frontier in lower- and higher-SDI settings, as well as the countries farthest from the frontier, to identify settings with relatively efficient control and those requiring strengthened HTN-T2DN prevention and management efforts.

All GBD analyses were performed using R software (version 4.4.1).

## 3. Results

### 3.1. Results of MR

#### 3.1.1. Statistical Analysis Results

In the two-sample MR analysis with HTN as the exposure and DN as the outcome, we identified 264 SNPs with an F-statistic > 10 after applying the screening steps described above (the mean F-statistic was 62.227, ranging from 26.947 to 296.442). Among these, SNPs directly associated with the outcome (rs2823139, rs1047891, rs12509595, rs77924615) and those linked to confounders such as smoking and alcohol consumption (rs74439044, rs1801253, rs1275988, rs3918226, rs13107325) were excluded.

The random-effects IVW method demonstrated a positive causal relationship between HTN and DN risk (OR = 4.219, 95% CI: 1.807–9.853, *p* = 0.001). Similarly, the fixed-effects IVW method indicated a positive causal association (OR = 4.219, 95% CI: 1.768–10.067, *p* = 0.001). The remaining four methods (MR-Egger, WM, WME, SM) yielded nonsignificant results (*p* > 0.05), though all showed ORs > 1, maintaining a consistent direction of effect (Table 2). Steiger directional testing confirmed the absence of reverse causality (*p* < 0.001). The BWMR model further reinforced a significant causal link between HTN and DN risk (*p* < 0.001, beta = 1.461, SE = 0.419) (Supplementary Figure S1).

#### 3.1.2. Sensitivity Analysis Results

Cochran’s Q tests revealed no heterogeneity (MR-Egger Q_pval = 0.676; IVW Q_pval = 0.692). The MR-Egger regression test for horizontal pleiotropy yielded *p* = 0.792 > 0.05, indicating that no horizontal pleiotropy was detected (Table 3). MR-PRESSO analysis found no evidence of pleiotropy or outlier SNPs. Convergent evidence from multiple methods suggests minimal confounding influence and high robustness of the results. These findings also guided the selection of the IVW fixed-effects model as the primary reference.

Scatter plots showed that the regression lines for the effect of HTN on DN were directionally consistent across methods. Funnel plots were essentially symmetric, and leave-one-out analyses indicated that no single SNP materially influenced the overall results (Supplementary Figure S2).

### 3.2. Results of GBD Analysis

#### 3.2.1. Global Overview of HTN-T2DN Disease Burden

Globally, deaths increased from 14,451 (95% UI: 2197 to 31,206) in 1990 to 50,689 (95% UI: 5484 to 115,116) in 2021. Similarly, DALYs rose from 382,969 (95% UI: 62,199 to 802,202) in 1990 to 1,151,216 (95% UI: 129,632 to 2,626,690) in 2021. From 1990 to 2021, both the ASMR and ASDR increased. The ASMR rose from 0.41 (95% UI: 0.06 to 0.91) per 100,000 population in 1990 to 0.61 (95% UI: 0.06 to 1.38) per 100,000 in 2021, while the ASDR increased from 10.19 (95% UI: 1.67 to 21.29) to 13.36 (95% UI: 1.50 to 30.52) per 100,000 over the same period.

**Figure 2:**
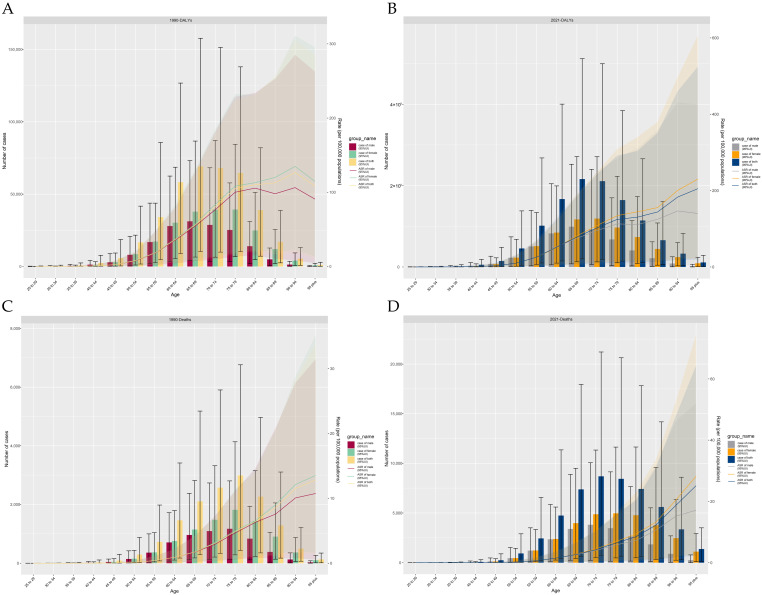
HTN-T2DN cases and age-standardized rates by sex and age group in 1990 and 2021. (**A**) DALYs in 1990; (**B**) DALYs in 2021; (**C**) deaths in 1990; (**D**) deaths in 2021. HTN-T2DN, type 2 diabetic nephropathy attributable to hypertension; DALYs, disability-adjusted life years; ASMR, age-standardized mortality rate; ASDR, age-standardized DALY rate.

In 2021, females exhibited higher ASMR and ASDR than males. The ASMR for females was 0.64 (95% UI: 0.08 to 1.39) compared to 0.55 (95% UI: 0.05 to 1.36) for males, while the ASDR for females was 13.98 (95% UI: 1.73 to 30.17) versus 12.51 (95% UI: 1.13 to 29.94) for males (Figure 2, Supplementary Table S1 (Sheet S1)). Age-stratified analysis revealed that both ASMR and ASDR increased with advancing age. In 2021, the highest ASMR and ASDR were observed in those aged 95 years and older, reaching 25.10 (95% UI: 2.27 to 64.37) and 205.00 (95% UI: 19.10 to 524.77), respectively.

**Figure 3:**
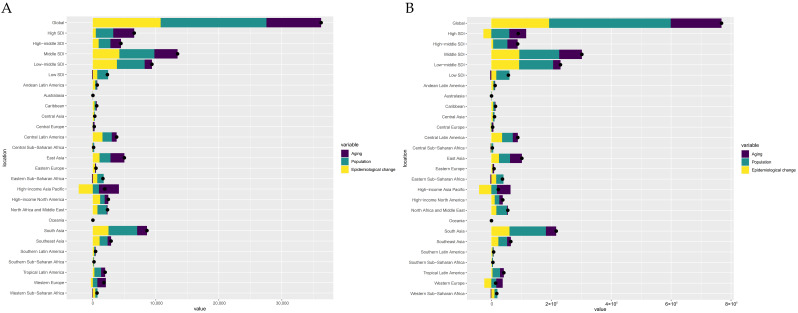
Changes in deaths (**A**) and DALYs (**B**) according to population growth, aging, and epidemiological change from 1990 to 2021 at the global level and across SDI regions. HTN-T2DN, type 2 diabetic nephropathy attributable to hypertension; DALYs, disability-adjusted life years; SDI, sociodemographic index.

At the GBD regional level, South Asia recorded the highest number of deaths in 2021 (11,227 deaths; 95% UI: 1282 to 23,420), whereas East Asia had the highest DALY burden (132,666 DALYs; 95% UI: 365 to 431,214). Central Latin America exhibited the highest ASMR (1.80; 95% UI: 0.26 to 3.51) and ASDR (41.33; 95% UI: 6.22 to 78.27). Among SDI regions, the low-middle SDI region showed the highest ASMR (0.92; 95% UI: 0.10 to 1.99) and ASDR (21.41; 95% UI: 2.14 to 46.22), while the high-middle SDI group had the lowest rates (ASMR: 0.35; 95% UI: 0.03 to 0.84; ASDR: 7.87; 95% UI: 0.82 to 18.48). Notably, high and high-middle SDI regions demonstrated significantly lower disease burdens than middle, low, and low-middle SDI regions, suggesting that HTN-T2DN disproportionately affected areas with lower socioeconomic development. Decomposition analysis of GBD regions indicated that global increases in HTN-T2DN burden were driven by population growth, population aging, and epidemiological shifts, with population growth contributing most substantially. In contrast, high-SDI regions (e.g., High-income Asia Pacific and Western Europe) experienced reduced burdens due to favorable epidemiological changes (Figure 3, Supplementary Table S1 (Sheet S2)).

**Figure 4:**
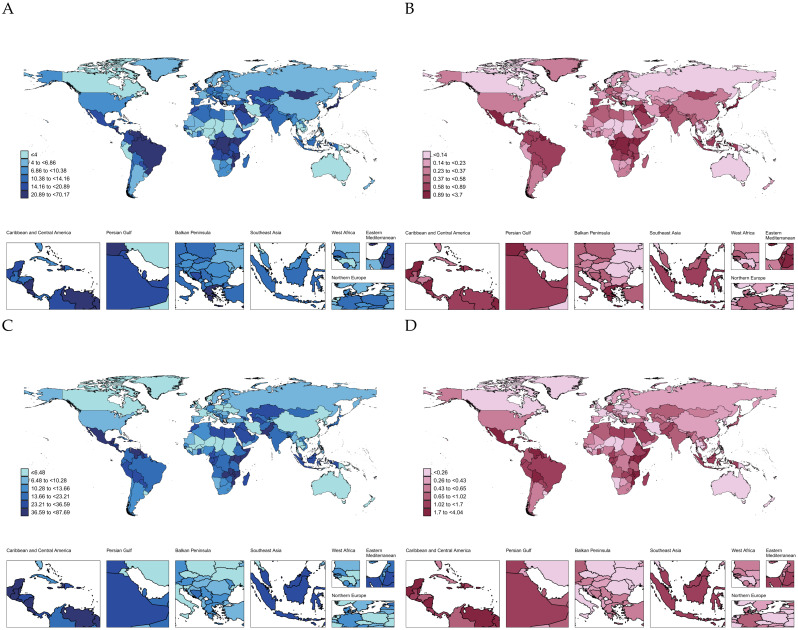
Global distribution of HTN-T2DN burden across countries and territories in 1990 and 2021. (**A**) ASDR in 1990; (**B**) ASMR in 1990; (**C**) ASDR in 2021; (**D**) ASMR in 2021. HTN-T2DN, type 2 diabetic nephropathy attributable to hypertension; DALYs, disability-adjusted life years; ASMR, age-standardized mortality rate; ASDR, age-standardized DALY rate; AAPC, average annual percent change.

At the national level, the five countries/territories with the highest ASMR in 2021 were the Republic of Trinidad and Tobago, Republic of Malawi, Republic of South Sudan, Saint Lucia, and Republic of Uganda. The lowest ASMR values were observed in the Independent State of Papua New Guinea, Ukraine, Australia, Republic of Tajikistan, and Republic of Belarus. For ASDR, the highest rates were recorded in Hungary, Republic of Albania, Islamic Republic of Mauritania, Slovak Republic, and Saint Kitts and Nevis, while the lowest rates occurred in the Independent State of Papua New Guinea, Canada, Republic of Guinea, Republic of Iceland, and Kingdom of Cambodia (Figure 4, Supplementary Table S1 (Sheet S3)).

#### 3.2.2. Temporal Trends in HTN-T2DN Disease Burden

Globally, the AAPCs for deaths and DALYs from 1990 to 2021 were 1.24 (95% CI: 1.21 to 1.27) and 0.88 (95% CI: 0.87 to 0.90), respectively, indicating an upward trajectory. The growth rates of ASMR and ASDR varied over time: ASMR growth decelerated markedly between 2001 and 2008, whereas ASDR declined from 2000 to 2009, suggesting a temporary period of improved control. However, both ASMR and ASDR resumed growth after 2009, highlighting renewed challenges in HTN-T2DN prevention and management. Females exhibited higher AAPC values than males, indicating a greater increase in burden among women.

**Figure 5:**
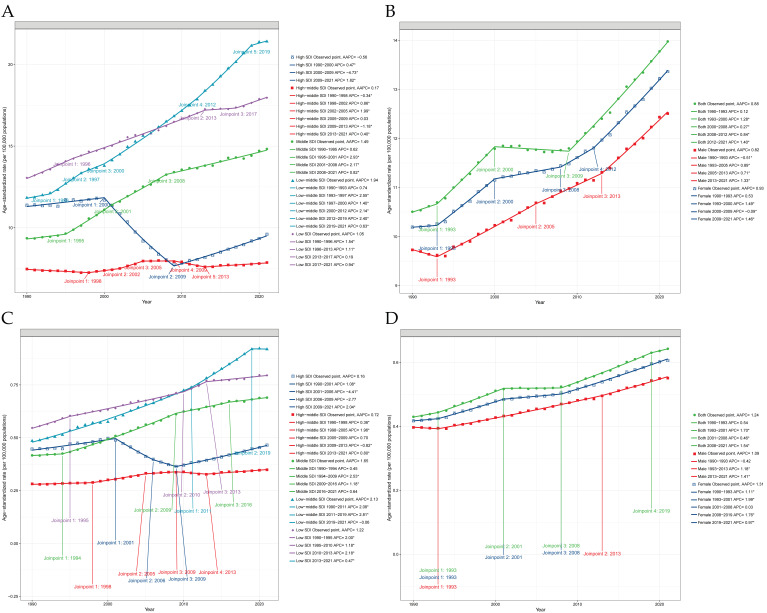
Trends of HTN-T2DN by sex and SDI region from 1990 to 2021, calculated by Joinpoint regression. (**A**) ASDR by SDI group; (**B**) ASDR by sex; (**C**) ASMR by SDI group; (**D**) ASMR by sex. Note: * indicates that the APC is significantly different from zero at *p* < 0.05. HTN-T2DN, type 2 diabetic nephropathy attributable to hypertension; DALYs, disability-adjusted life years; AAPC, average annual percent change; APC, annual percent change.

Across SDI regions, ASMR increased universally, with the fastest growth in the low-middle SDI region (AAPC: 2.13; 95% CI: 2.06 to 2.18) and the slowest in the high-SDI region (AAPC: 0.16; 95% CI: 0.11 to 0.21). The high-SDI region demonstrated a pronounced decline in ASMR from 2001 to 2009. Similarly, the high-middle SDI region showed the smallest ASDR growth (AAPC: 0.17; 95% CI: 0.15 to 0.19), whereas high-SDI regions experienced negative growth (AAPC: −0.56; 95% CI: −0.60 to −0.51), with significant reductions in ASDR between 2000 and 2009, indicative of successful HTN-T2DN mitigation strategies in these areas (Figure 5).

#### 3.2.3. Projections of HTN-T2DN Disease Burden for 2022–2045

**Figure 6:**
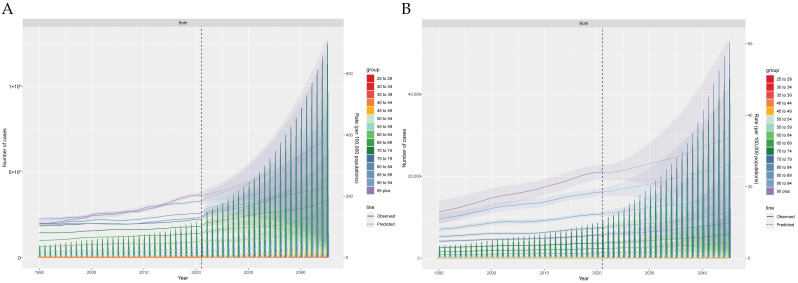
Projected HTN-T2DN burden by age from 2022 to 2045. (**A**) DALYs by age; (**B**) deaths by age. HTN-T2DN, type 2 diabetic nephropathy attributable to hypertension; DALYs, disability-adjusted life years.

From 2022 to 2045, deaths, DALYs, ASMR, and ASDR were projected to rise significantly. By 2045, HTN-T2DN deaths and DALYs were estimated to reach 162,392 (95% UI: 31,129 to 293,754) and 4,041,984 (95% UI: 681,834 to 7,402,564), respectively. Global ASMR and ASDR were predicted to climb to 0.96 (95% UI: 0.18 to 1.74) and 25.40 (95% UI: 4.32 to 46.48) (Figure 6).

**Figure 7:**
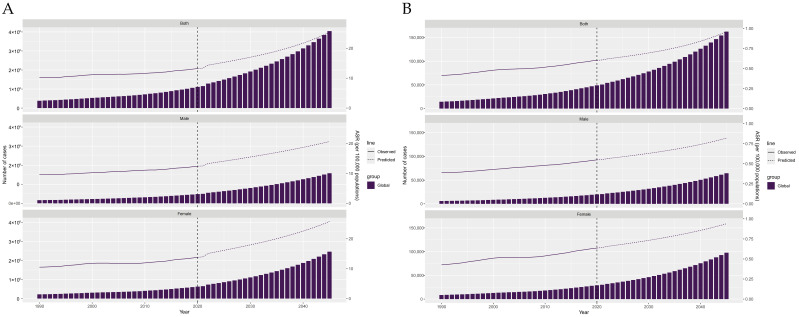
Projected HTN-T2DN burden by sex from 2022 to 2045. (**A**) DALYs by sex; (**B**) deaths by sex. HTN-T2DN, type 2 diabetic nephropathy attributable to hypertension; DALYs, disability-adjusted life years.

Sex disparities were expected to widen, with females sustaining higher ASMR and ASDR than males. By 2045, people aged 95 years and older were projected to bear the highest burden (ASMR: 33.58; 95% UI: 5.67 to 61.49; ASDR: 233.89; 95% UI: 111.78 to 436.28), whereas the 25-to-29-year age group had the lowest rates (ASMR: <0.01; ASDR: 0.05; 95% UI: 0.01 to 0.14), emphasizing the need for targeted interventions for elderly populations (Figure 7).

#### 3.2.4. Frontier Analysis of HTN-T2DN Disease Burden

**Figure 8:**
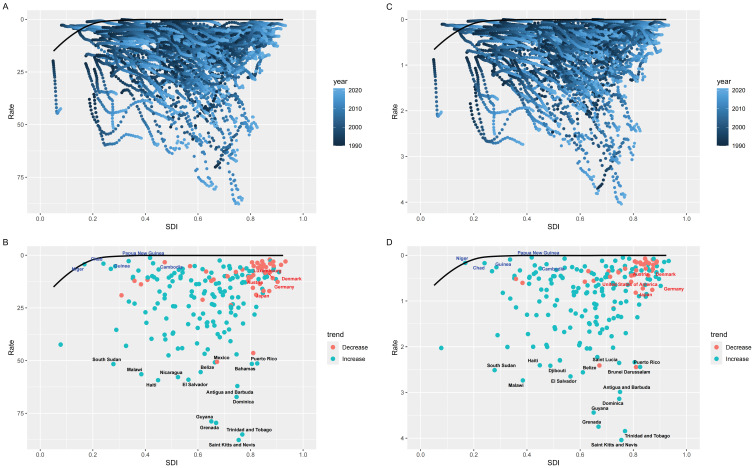
Frontier analysis based on SDI and HTN-T2DN burden across 204 countries and territories. (**A**) ASDR by year; (**B**) ASDR across countries and territories; (**C**) ASMR by year; (**D**) ASMR across countries and territories. HTN-T2DN, type 2 diabetic nephropathy attributable to hypertension; SDI, sociodemographic index; ASMR, age-standardized mortality rate; ASDR, age-standardized DALY rate.

Frontier analysis identified countries deviating furthest from optimal HTN-T2DN management relative to their SDI. For ASMR, Saint Kitts and Nevis, Trinidad and Tobago, Grenada, Guyana, and Dominica exhibited the largest gaps. Among high-SDI nations, Austria, Denmark, the United States of America, Germany, and Japan performed closest to the efficiency frontier. In low-SDI countries, Papua New Guinea, Guinea, Niger, Chad, and Cambodia aligned most closely with benchmarked outcomes. Similar trends were observed for ASDR, with Saint Kitts and Nevis, Trinidad and Tobago, Grenada, Guyana, and Dominica showing the greatest disparities. High-SDI leaders included Luxembourg, Denmark, Austria, Germany, and Japan, while low-SDI performers mirrored the ASMR results. Countries farther from the frontier require intensified HTN-T2DN interventions, whereas those near the frontier offer models for effective management (Figure 8).

## 4. Discussion

The MR analysis provided genetic evidence supporting a potential positive association between HTN and DN (IVW OR = 4.219, *p* = 0.001). Rather than being interpreted as a precise clinical risk estimate, this result should be viewed as supportive evidence that genetically predicted HTN may increase susceptibility to DN. The GBD analysis demonstrated a 3.5-fold increase in HTN-T2DN-related deaths and a threefold rise in DALYs from 1990 to 2021. Women and older adults exhibited disproportionately higher burdens, with ASMR and ASDR in low-middle-SDI regions (e.g., South Asia, Central Latin America) substantially exceeding those in high-SDI areas. Despite transient deceleration in disease progression during 2000–2009, post-2009 trends resumed an upward trajectory. Projections based on historical patterns suggest that deaths and DALYs may reach approximately 160,000 and 4.04 million by 2045. These findings indicate that comorbid HTN and diabetes-related kidney disease require sustained clinical and public health attention.

Several aspects of the MR findings require cautious interpretation. The outcome GWAS included 1032 DN cases and 451,248 controls; this small number of cases may have reduced statistical power and contributed to the wide confidence interval around the IVW estimate. Although the IVW and BWMR analyses reached statistical significance and the other MR methods showed a consistent direction of effect, MR-Egger, weighted median, simple mode, and weighted mode did not reach statistical significance. These sensitivity analyses should therefore be viewed as supportive assessments of robustness rather than definitive exclusion of pleiotropy or bias. Similarly, non-significant tests for heterogeneity, the MR-Egger intercept, and MR-PRESSO suggest no statistical evidence of major violations, but they cannot completely rule out subtle horizontal pleiotropy, phenotype heterogeneity, or residual bias.

Diabetic nephropathy is common in people with diabetes, and hypertension accelerates renal function decline and worsens prognosis in patients with diabetic kidney disease [31,32,33]. The coexistence of hypertension and diabetes has also been associated with greater multiorgan vulnerability in acute settings, including COVID-19 [34]. These observations are consistent with our MR findings and with the growing population-level burden identified in the GBD analysis.

Several biological pathways may help explain the relationship between hypertension and T2DN, including activation of the renin–angiotensin–aldosterone system, abnormalities in sodium handling, oxidative stress, inflammation, and glomerular hemodynamic changes [31,35,36,37,38,39,40]. Recent therapeutic advances, particularly sodium-glucose cotransporter 2 inhibitors and non-steroidal mineralocorticoid receptor antagonists, have shown renoprotective benefits that extend beyond conventional ACEI/ARB-based care [35,36,37,38,39,40].

Although the medical community has achieved substantial progress in prevention and treatment in recent years, this GBD analysis revealed a striking global increase in the disease burden of HTN-T2DN over the past three decades. This burden particularly affects elderly populations, likely owing to cumulative vascular damage, declining renal functional reserve, and polypharmacy commonly observed in this demographic. A survey of diabetic patients in Ethiopia reported a DN prevalence of 31.5% and identified advanced age (≥60 years) and comorbid HTN as the primary risk factors (*p*-values of 0.003 and 0.043, respectively) [41]. The decline in renal function among DN patients was positively correlated with SBP and proteinuria levels, and this association was particularly pronounced in the elderly population [42].

Geographically, South Asia and Central Latin America emerged as hotspots for HTN-T2DN mortality and DALY burden, potentially reflecting rising diabetes prevalence, uneven access to antihypertensive treatment, and disparities in healthcare resources. Evidence from South Asia and from evaluations of primary healthcare infrastructure in the region supports the importance of strengthening early diagnosis and chronic disease management capacity [43,44]. By contrast, high-income Asia Pacific and Western Europe showed lower or declining burdens, which may reflect stronger health systems, earlier screening, and more effective blood pressure control. Our decomposition analyses further suggested that population growth was a major driver of increasing global DALYs, whereas epidemiological improvements helped mitigate burden growth in high-SDI regions [45].

Notably, significant sex disparities were observed, with higher ASMR and ASDR in females than in males. This finding contrasts sharply with traditional patterns of higher cardiovascular risk in males and suggests the existence of sex-specific factors in DN progression. Although estrogen can exert renal protective effects by suppressing the activity of the renin-angiotensin system, reducing oxidative stress, and mitigating fibrosis [46], under diabetic conditions, hyperglycemia may induce abnormal estrogen metabolism. For instance, postmenopausal women experience a sharp decline in estrogen levels, while diabetes accelerates the reduction in aromatase activity, further reducing estrogen-related conversion [47]. Other potential factors may include differences in healthcare-seeking behavior or underdiagnosis in men. This highlights the urgent need for further research to elucidate these mechanisms and develop sex-sensitive intervention strategies.

We estimated that the burden of HTN-T2DN could rise substantially by 2045, highlighting the need for coordinated global action; however, these forecasts should be interpreted as conditional projections rather than deterministic predictions. The BAPC model extrapolates from historical trends, age structure, and population dynamics, and future disease trajectories may be altered by improved blood pressure control, wider use of SGLT2 inhibitors or non-steroidal mineralocorticoid receptor antagonists, changes in diabetes prevalence, health-system reforms, or major public health disruptions. Population aging, particularly in low- and middle-income settings, is likely to intensify this challenge. Frontier analysis should also be understood as a benchmarking tool rather than a causal analysis. It identifies countries whose observed ASMR or ASDR is higher than the minimum achievable burden at comparable SDI levels, thereby suggesting potential opportunities for health-system improvement. For resource-constrained settings, feasible strategies include low-cost screening, strengthened primary care, better health literacy, and the use of mHealth-supported community interventions to improve chronic disease monitoring and follow-up [48]. Addressing the social determinants of health remains essential to reducing the marked disparities observed across SDI regions.

Although this study provides comprehensive insights, several limitations warrant attention. First, the MR analysis relied exclusively on European ancestry data, whereas the GBD analysis encompassed global populations. This discrepancy restricts generalizability, as genetic background, environmental exposures, lifestyle patterns, and healthcare access may alter causal effect sizes across diverse ethnic groups. Therefore, caution is warranted when extrapolating European-derived MR estimates to a global context; the GBD component should be regarded as a descriptive and forecasting analysis of global burden rather than evidence that the same genetic effect applies uniformly across all populations. Second, although T2DM accounts for approximately 90–95% of global diabetes cases [49], the DN phenotype in the GWAS dataset did not strictly distinguish between diabetes types. Consequently, the potential inclusion of type 1 diabetes-related nephropathy or heterogeneous DN definitions may affect the specificity of the causal inference. Third, the number of DN cases in the outcome GWAS was relatively small, which may reduce statistical power and precision and may partially explain the wide confidence intervals of the MR estimates. Fourth, we acknowledge substantial sample overlap because both exposure and outcome datasets were sourced from the UK Biobank. Sample overlap can bias MR estimates towards observational associations, particularly when instruments are weak. Although all retained instruments had F-statistics > 10 and sensitivity analyses did not identify major heterogeneity or directional pleiotropy, these approaches cannot fully eliminate the potential influence of overlap. Finally, the GBD dataset relies on modeled estimates, introducing uncertainties in data-sparse regions such as sub-Saharan Africa. Misclassification of DN etiology and underreporting in resource-limited areas could further bias burden assessments. The predictive model also assumes that historical demographic and epidemiological patterns will continue and may not fully capture emerging technologies, novel therapies such as SGLT2 inhibitors, or societal disruptions such as pandemics.

Future studies should aim to incorporate independent datasets with larger numbers of DN cases, stricter phenotype definitions that distinguish between diabetes types, and broader ancestry representation to validate these findings and reduce sample-overlap concerns. Specifically, once the complete GBD 2023 dataset becomes publicly available, subsequent research is warranted to update these epidemiological trends and capture the latest global shifts. Furthermore, dynamic prediction models should be developed that integrate variables such as environmental exposures, the impact of novel pharmaceutical interventions, and major public health events. Strengthening epidemiological surveillance in data-sparse regions remains essential to enhance the precision and adaptability of disease burden estimates. Finally, deeper investigation into the underlying molecular mechanisms of comorbidity between HTN and T2DM is critical to support the development of more targeted therapeutic interventions.

## 5. Conclusions

Using MR analysis, this study provided genetic evidence supporting a potential causal role for HTN in the development of DN. HTN was associated with an increased risk of DN (OR = 4.219, *p* = 0.001). An analysis of the 2021 GBD data revealed a global increase in HTN-T2DN burden between 1990 and 2021. The burden was most pronounced in low-SDI regions, women, and older adults, driven primarily by population growth and aging. Forecasts to 2045 predicted continued increases unless comprehensive interventions were adopted. Despite its limitations, our study highlighted the importance of blood pressure management in patients with T2DM. Future studies should use additional datasets to improve the generalizability of our findings. We also recommend that high-burden regions adopt effective approaches such as early screening and patient education to reduce the risk of renal disease associated with HTN in patients with T2DM.

## Figures and Tables

**Table 1:** Detailed information on the GWAS datasets used for HTN and DN.

Disease	IEU ID	Sample Size (Cases; Controls)	Number of SNPs	Year	Population	PubMed ID
HTN	GCST90038604	484,598 (129,909; 354,689)	9,587,836	2021	European	33959723
DN	GCST90018832	452,280 (1032; 451,248)	24,190,738	2021	European	34594039

**Table 2:** Mendelian randomization results for the effect of HTN on DN.

Outcome	Exposure	Method	nSNP	b	SE	*p*-Value	OR	OR_lci95	OR_uci95
DN	HTN	IVW (multiplicative random effects)	230	1.4396	0.4327	0.0009	4.2190	1.8066	9.8526
DN	HTN	IVW (fixed effects)	230	1.4396	0.4437	0.0012	4.2190	1.7681	10.0674
DN	HTN	MR-Egger	230	1.7425	1.2325	0.1588	5.7116	0.5101	63.9502
DN	HTN	Weighted median	230	1.1598	0.6812	0.0886	3.1894	0.8393	12.1204
DN	HTN	Simple mode	230	1.2138	1.8161	0.5046	3.3663	0.0958	118.3228
DN	HTN	Weighted mode	230	1.0800	1.4017	0.4418	2.9446	0.1887	45.9376

**Table 3:** Results of heterogeneity and horizontal pleiotropy analyses.

**Cochran’s Q Test for Heterogeneity Analysis**					
**Outcome**	**Exposure**	**Method**	**Q**	**Q_df**	**Q_pval**
DN	HTN	MR-Egger	217.7139	228	0.6764
DN	HTN	IVW	217.7833	229	0.6920
**MR-Egger regression horizontal pleiotropy test**					
**Outcome**	**Exposure**	**Egger_intercept**	**SE**	***p*** **-value**	
DN	HTN	−0.0025	0.0095	0.7925	

## Data Availability

Publicly available datasets were analyzed in this study. GWAS summary statistics were obtained from IEU OpenGWAS and the GWAS Catalog, and GBD 2021 estimates were obtained from the IHME GBD Results Tool. The code and Supplementary Materials used in this study are available at https://github.com/HDS-GO/Causal-Relationship-Between-Hypertension-and-Type-2-Diabetic-Nephropathy (accessed on 8 February 2026).

## Supplementary Materials

The following supporting information can be downloaded at: https://www.mdpi.com/article/10.3390/healthcare14121725/s1, STROBE-MR checklist; Table S1: sheets S1–S3; Figure S1: Diagnostic plots from the Bayesian weighted Mendelian randomization (BWMR) analysis evaluating the causal effect of genetically predicted hypertension on diabetic nephropathy. (Plot 1) Scatter plot of SNP–exposure associations against SNP–outcome associations with corresponding standard error bars for each instrumental variant. (Plot 2) Trajectory of the evidence lower bound (ELBO) across variational iterations, showing rapid improvement followed by stabilization, indicative of satisfactory model convergence. (Plot 3) Posterior mean weight assigned to each observation (instrumental SNP); most variants received weights close to 1.0, whereas a small number were down-weighted, suggesting potential outlying or pleiotropic instruments. (Plot 4) Weighted regression of SNP–outcome associations on SNP–exposure associations. Point color represents the posterior weight assigned to each SNP, and the dashed line denotes the BWMR-estimated causal effect. Abbreviations: BWMR, Bayesian weighted Mendelian randomization; ELBO, evidence lower bound; SNP, single-nucleotide polymorphism; Figure S2: Sensitivity analyses for the Mendelian randomization estimate of the causal effect of genetically predicted hypertension on diabetic nephropathy. (Plot 1) Forest plot of single-SNP causal estimates together with the pooled inverse-variance weighted (IVW) and MR-Egger estimates. Black points represent SNP-specific estimates, horizontal lines denote 95% confidence intervals, and red points indicate the pooled estimates. (Plot 2) Funnel plot of single-SNP causal estimates (β_IV) against their precision (1/SE_IV). Vertical lines represent the IVW and MR-Egger estimates and were used to assess asymmetry suggestive of directional pleiotropy. (Plot 3) Leave-one-out sensitivity analysis showing the IVW estimate obtained after sequential exclusion of each SNP; the red point and horizontal line denote the overall estimate derived from all SNPs, indicating that the association was not materially driven by any single variant. (Plot 4) Scatter plot of SNP-specific associations with fitted slopes from IVW fixed-effects, IVW multiplicative random-effects, MR-Egger, weighted median, weighted mode, and simple mode methods. Black dots represent individual SNPs, and error bars indicate standard errors of the SNP-specific associations. Abbreviations: IVW, inverse-variance weighted; MR, Mendelian randomization; SNP, single-nucleotide polymorphism.
