# Supplementary material for: Potential Causal Relationship Between Hypertension and Type 2 Diabetic Nephropathy: Integrating Mendelian Randomization Evidence with Global Burden of Disease 2021 Analysis
Source: Healthcare (Basel). 2026 Jun 15;14(12):1725. doi: 10.3390/healthcare14121725 (PMC13299940; doi:10.3390/healthcare14121725)
Supplement: Supplementary file 1 [file healthcare-14-01725-s001.zip › Supplementary Figure S1.pdf]

**Supplementary Figure S1**

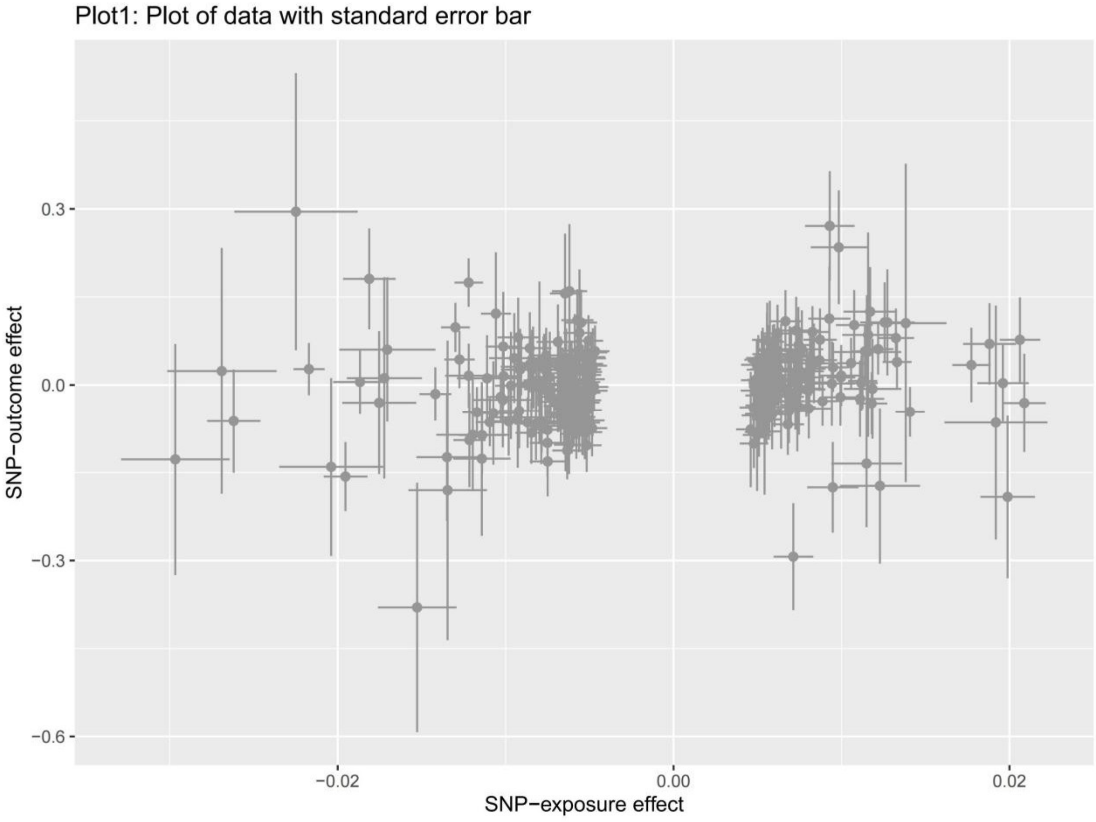

Plot2: Plot of evidence lower bound (elbo)

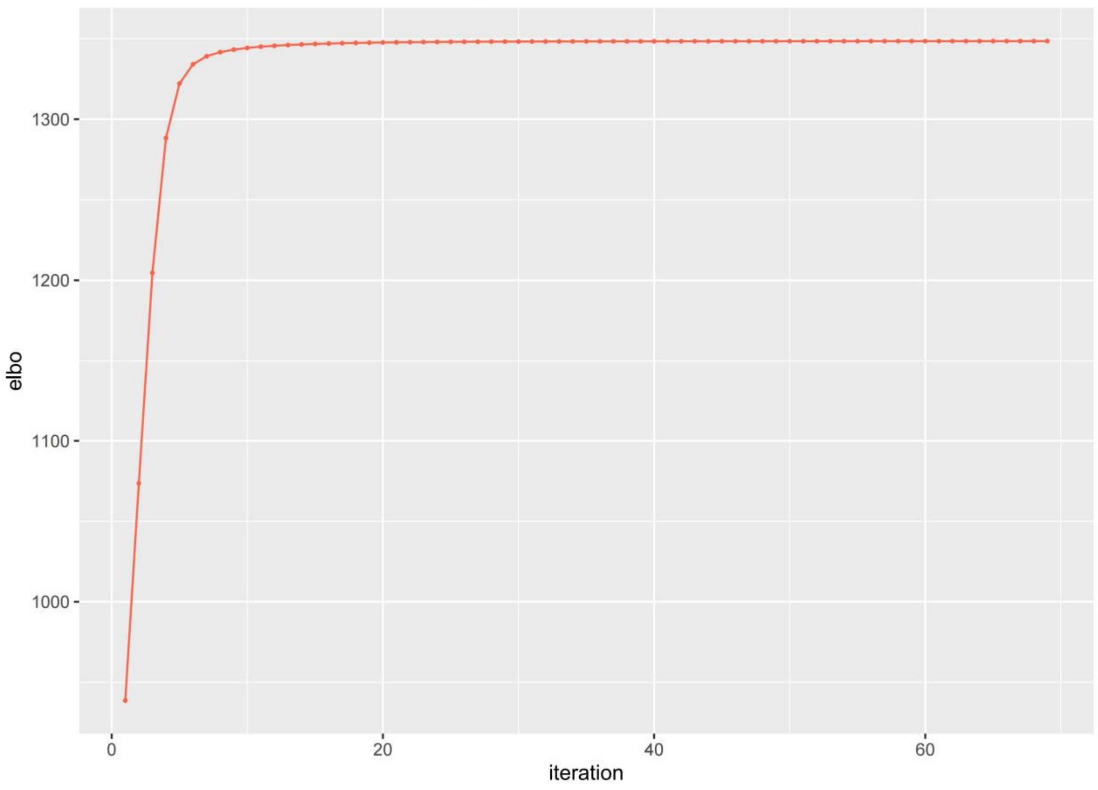


Plot3: Posterior mean of weight of each observation

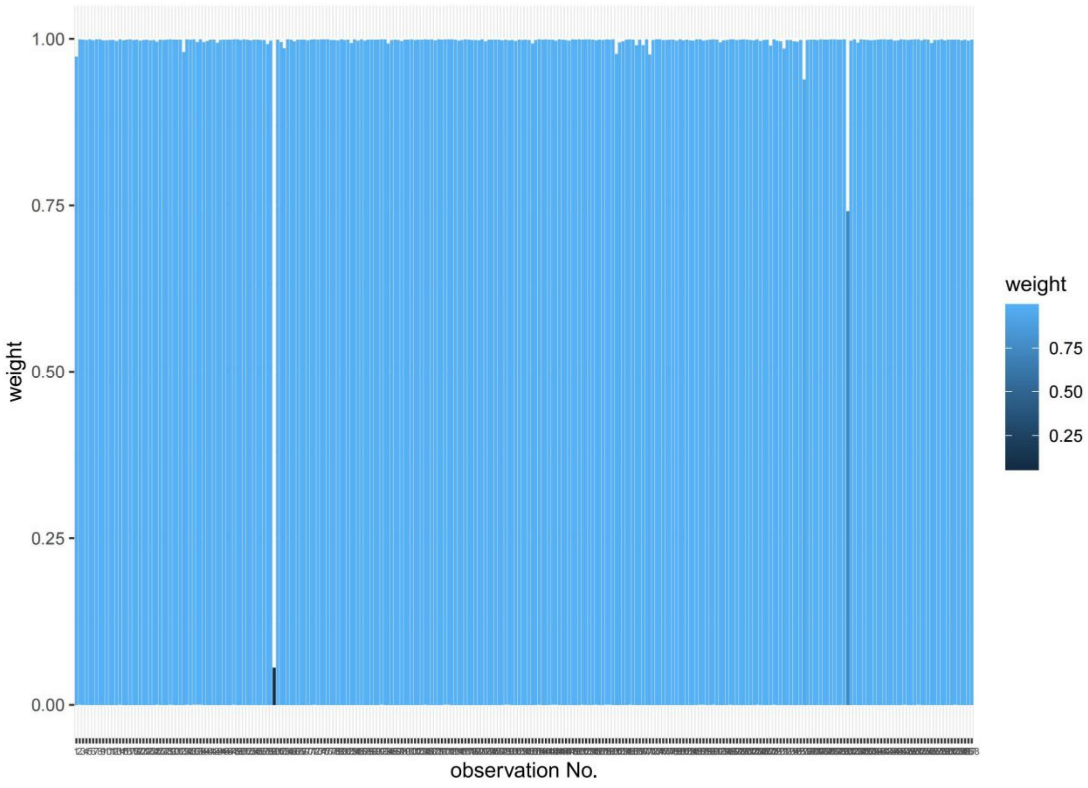

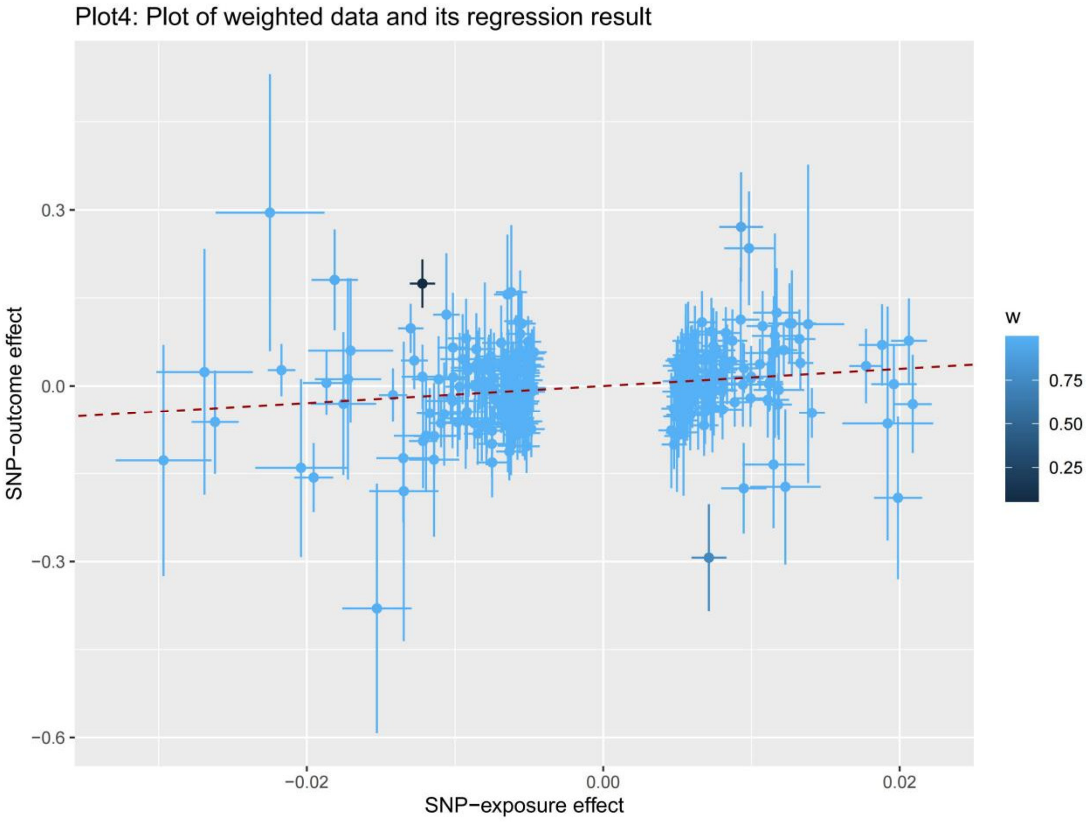


**Supplementary Figure S1.** Diagnostic plots from the Bayesian weighted Mendelian randomization (BWMR) analysis evaluating the causal effect of genetically predicted hypertension on diabetic nephropathy. (Plot 1) Scatter plot of SNP–exposure associations against SNP－outcome associations with corresponding standard error bars for each instrumental variant. (Plot 2) Trajectory of the evidence lower bound (ELBO) across variational iterations, showing rapid improvement followed by stabilization, indicative of satisfactory model convergence. (Plot 3) Posterior mean weight assigned to each observation (instrumental SNP); most variants received weights close to 1.0, whereas a small number were down-weighted, suggesting potential outlying or pleiotropic instruments. (Plot 4) Weighted regression of SNP－outcome associations on SNP–exposure associations. Point color represents the posterior weight assigned to each SNP, and the dashed line denotes the BWMR-estimated causal effect. Abbreviations: BWMR, Bayesian weighted Mendelian randomization; ELBO, evidence lower bound; SNP, single-nucleotide

polymorphism.
