# Supplementary material for: Potential Causal Relationship Between Hypertension and Type 2 Diabetic Nephropathy: Integrating Mendelian Randomization Evidence with Global Burden of Disease 2021 Analysis
Source: Healthcare (Basel). 2026 Jun 15;14(12):1725. doi: 10.3390/healthcare14121725 (PMC13299940; doi:10.3390/healthcare14121725)
Supplement: Supplementary file 1 [file healthcare-14-01725-s001.zip › Supplementary Figure S2.pdf]

Supplementary Figure S2

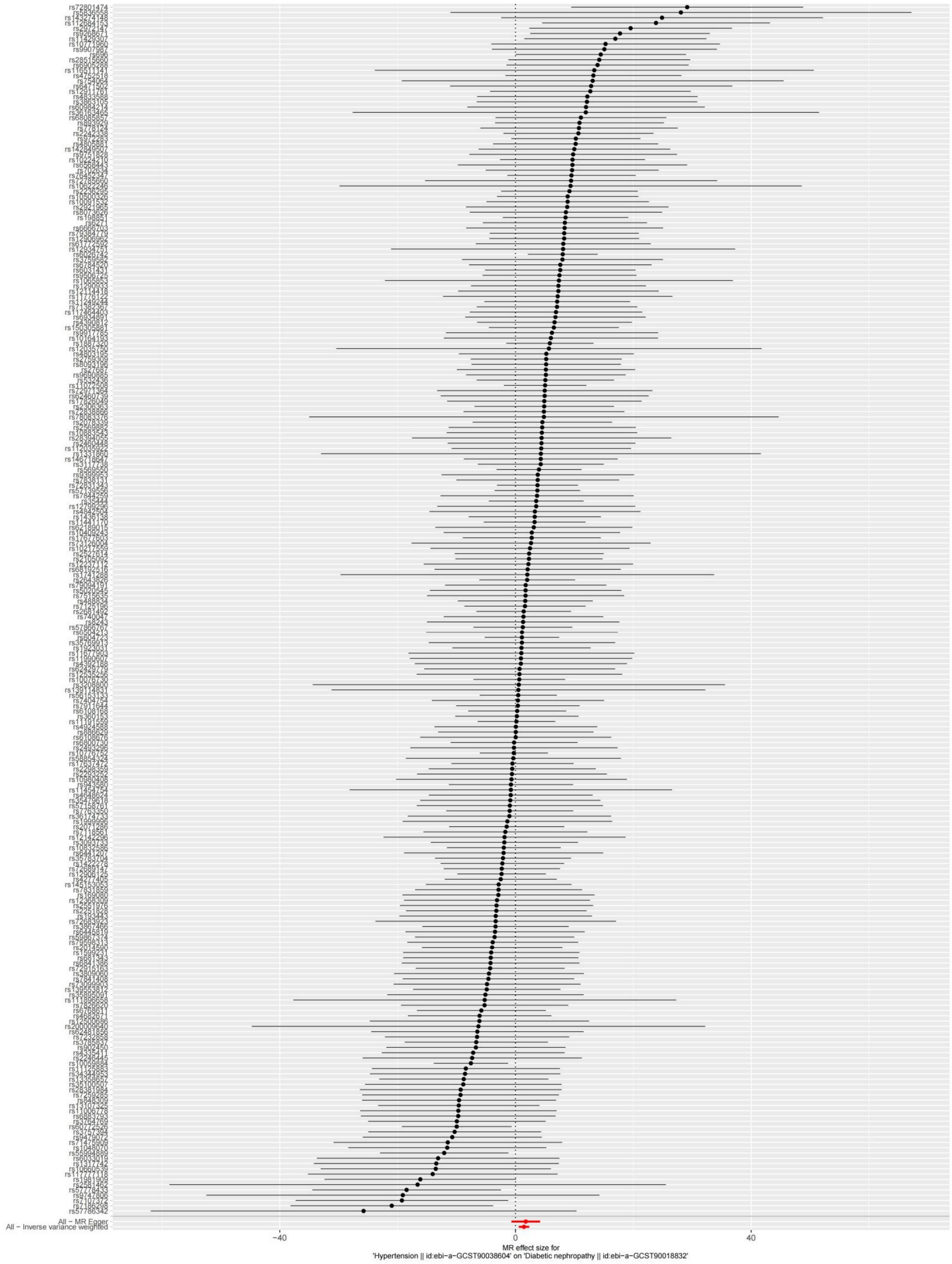

Plot 1: SNPs forest plot

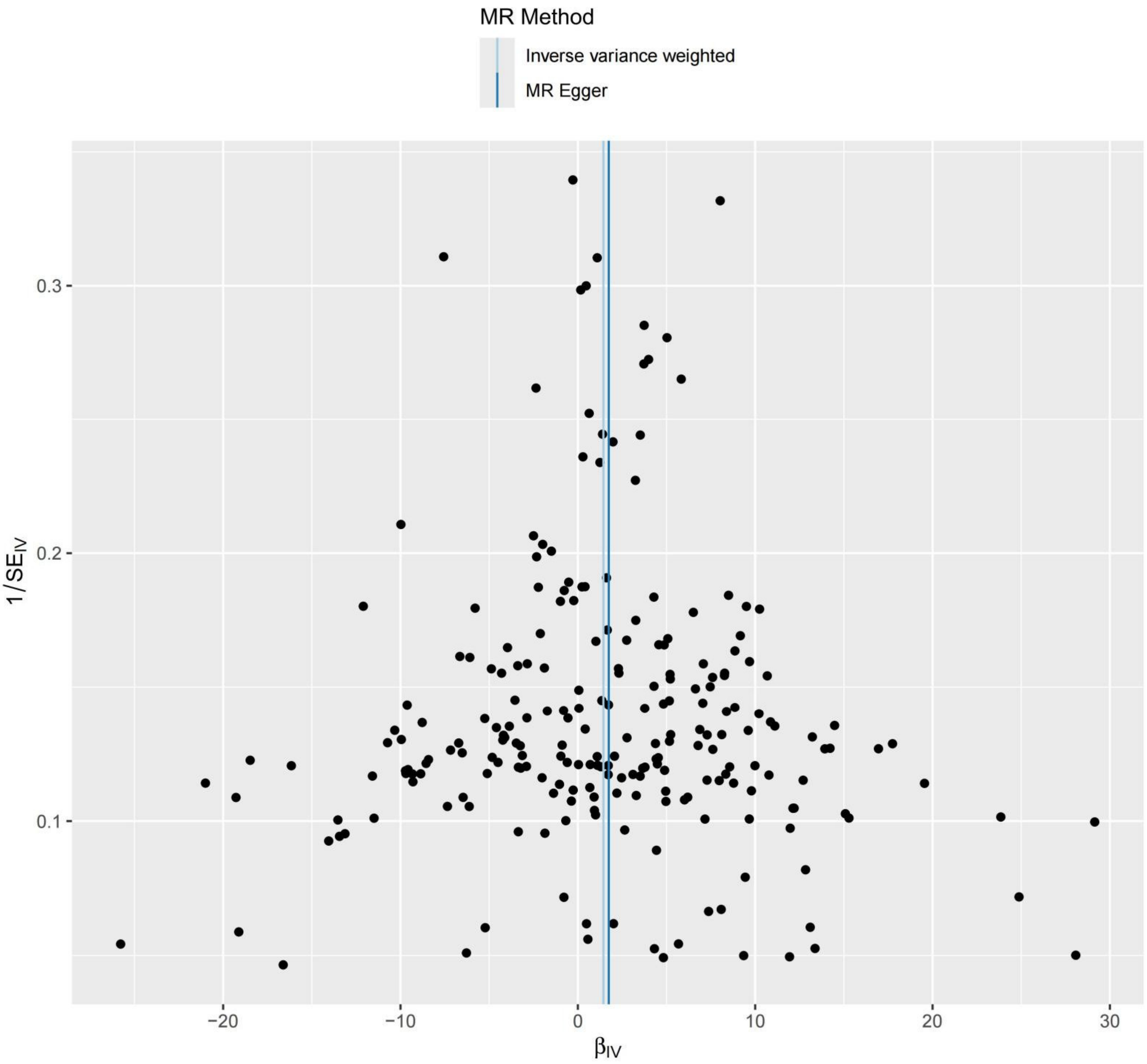

Plot 2: Funnel plot

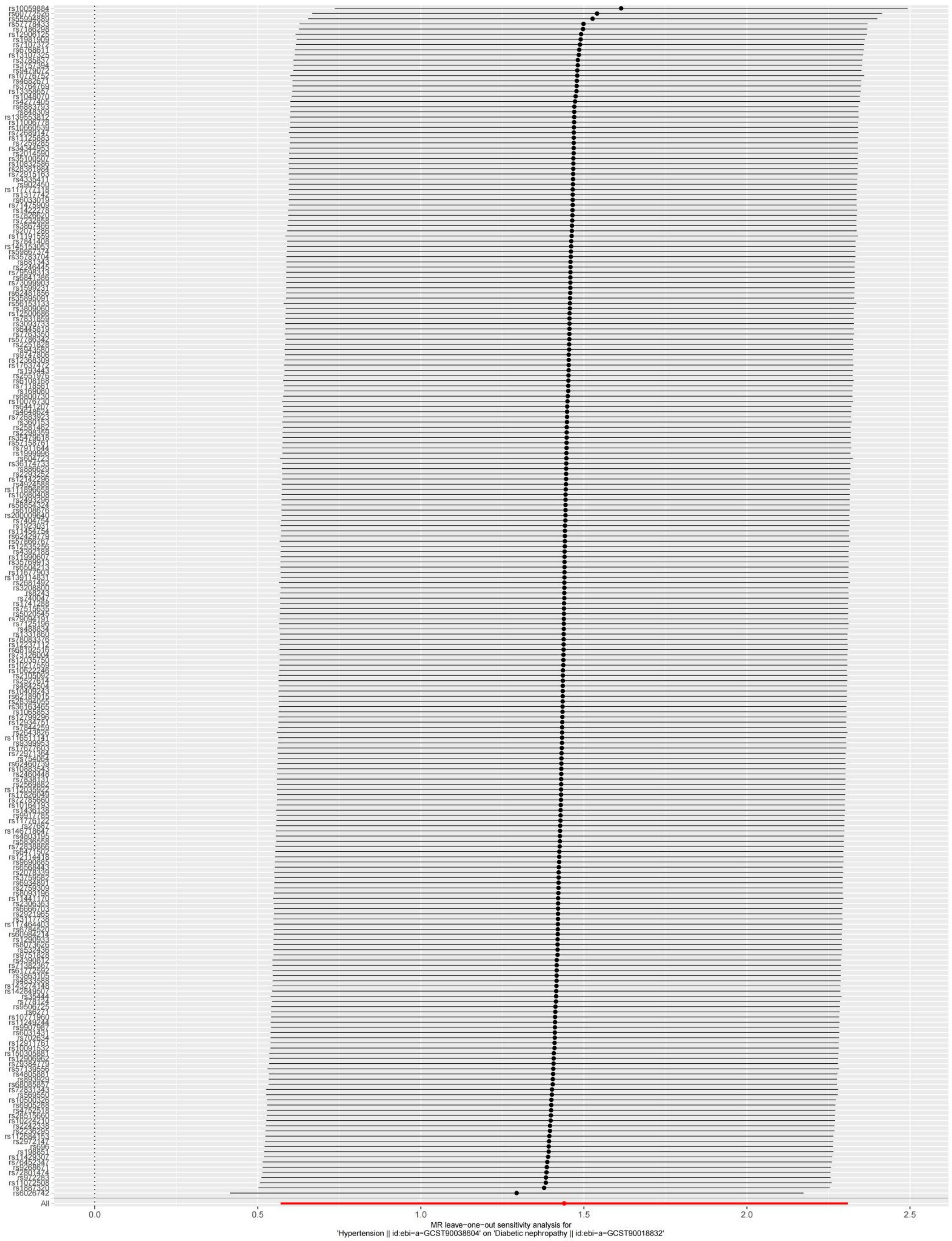

Plot 3: Leave-one-out analysis results

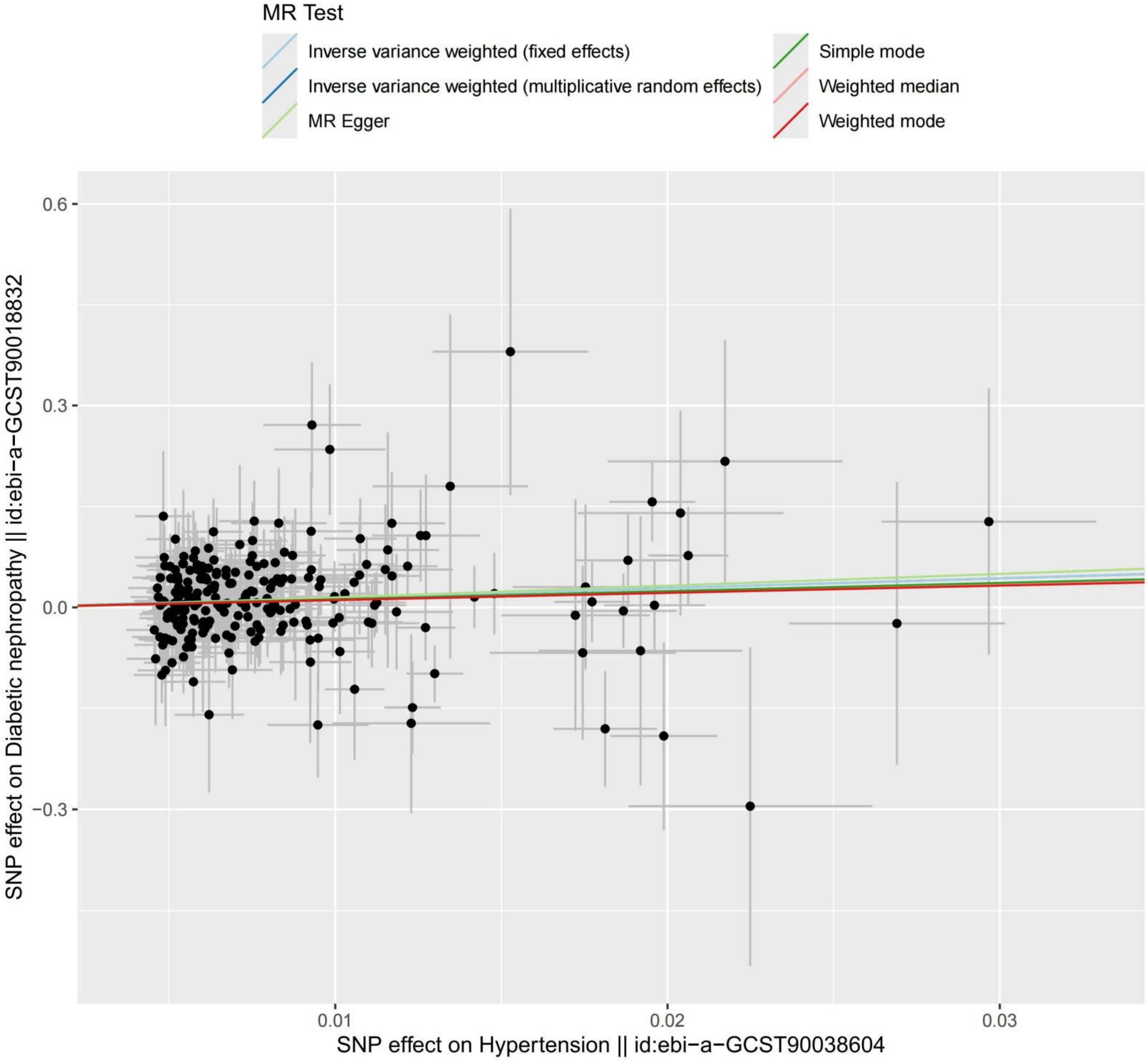

Plot 4: Scatter plot

Supplementary Figure S2. Sensitivity analyses for the Mendelian randomization estimate of the causal effect of genetically predicted hypertension on diabetic nephropathy. (Plot 1) Forest plot of single-SNP causal estimates together with the pooled inverse-variance weighted (IVW) and MR-Egger estimates. Black points represent SNP-specific estimates, horizontal lines denote 95% confidence intervals, and red points indicate the pooled estimates. (Plot 2) Funnel plot of single-SNP causal estimates (β_IV) against their precision (1/SE_IV). Vertical lines represent the IVW and MR-Egger estimates and were used to assess asymmetry suggestive of directional pleiotropy. (Plot 3) Leave-one-out sensitivity analysis showing the IVW estimate obtained after sequential exclusion of each SNP; the red point and horizontal line denote the overall estimate derived from all SNPs, indicating that the association was not materially driven by any single variant. (Plot 4) Scatter plot of SNP-specific associations with fitted slopes from IVW fixed-effects, IVW

multiplicative random-effects, MR-Egger, weighted median, weighted mode, and simple mode methods. Black dots represent individual SNPs, and error bars indicate standard errors of the SNP-specific associations. Abbreviations: IVW, inverse-variance weighted; MR, Mendelian randomization; SNP, single-nucleotide polymorphism.
