# Supplementary material for: Potential Causal Relationship Between Hypertension and Type 2 Diabetic Nephropathy: Integrating Mendelian Randomization Evidence with Global Burden of Disease 2021 Analysis
Source: Healthcare (Basel). 2026 Jun 15;14(12):1725. doi: 10.3390/healthcare14121725 (PMC13299940; doi:10.3390/healthcare14121725)
Supplement: Supplementary file 1 [file healthcare-14-01725-s001.zip › Supplementary Table S1.pdf]

Supplementary Table S1

Sheet S1

	Deaths					DALYs				
	**CASE**	**ASMR**	**95%UI**	**AAPC**	**95%UI**	**CASE**	**ASMR**	**95%UI**	**AAPC**	**95%UI**
Global	50689	0.61	(0.06,1.38)	1.24	(1.21,1.27)	1151216	13.36	(1.50,30.52)	0.88	(0.87,0.9)
Sex										
Males	20515	0.55	(0.05,1.36)	1.09	(1.05,1.13)	498678	12.51	(1.13,29.94)	0.82	(0.8,0.85)
Females	30173	0.64	(0.08,1.39)	1.31	(1.28,1.35)	652538	13.98	(1.73,30.17)	0.93	(0.91,0.95)
SDI regions										
High SDI	11677	0.46	(0.07,1.06)	0.16	(0.11,0.21)	219280	9.60	(1.51,21.64)	-0.56	(-0.60,-0.51)
High-middle SDI	6839	0.35	(0.03,0.84)	0.72	(0.68,0.76)	158028	7.87	(0.82,18.48)	0.17	(0.15,0.19)
Low SDI	3368	0.79	(0.08,1.83)	1.22	(1.17,1.28)	84915	17.97	(1.90,40.65)	1.05	(1.01,1.09)
Low-middle SDI	11869	0.92	(0.10,1.99)	2.13	(2.06,2.18)	297769	21.41	(2.14,46.22)	1.94	(1.90,1.98)
Middle SDI	16884	0.69	(0.05,1.69)	1.65	(1.60,1.69)	389986	14.83	(1.25,35.43)	1.49	(1.45,1.54)
Age groups										
25 to 29	1	0.00	(<0.01,<0.01)	2.46	(2.42,2.52)	190	0.03	(<0.01,0.09)	0.37	(0.34,0.41)
30 to 34	6	0.00	(<0.01,<0.01)	1.66	(1.62,1.69)	597	0.10	(0.01,0.30)	0.47	(0.42,0.53)

35 to 39	19	0.00	(<0.01,0.01)	1.58	(1.52,1.63)	1614	0.29	(0.02,0.98)	0.75	(0.72,0.79)
40 to 44	77	0.02	(<0.01,0.06)	1.41	(1.37,1.44)	5333	1.07	(0.06,3.75)	0.75	(0.71,0.78)
45 to 49	247	0.05	(<0.01,0.19)	1.08	(1.02,1.15)	14600	3.08	(0.15,10.22)	0.64	(0.58,0.72)
50 to 54	923	0.21	(0.01,0.65)	1.32	(1.28,1.36)	45503	10.23	(0.66,30.97)	0.84	(0.81,0.87)
55 to 59	2445	0.62	(0.05,1.66)	1.51	(1.49,1.54)	101474	25.64	(2.48,67.74)	1.08	(1.05,1.11)
60 to 64	4734	1.48	(0.13,3.55)	1.61	(1.55,1.67)	166863	52.14	(4.65,125.15)	1.19	(1.15,1.23)
65 to 69	7370	2.67	(0.23,6.51)	1.52	(1.48,1.56)	215961	78.29	(7.28,185.70)	1.13	(1.10,1.15)
70 to 74	8689	4.22	(0.37,10.31)	1.06	(1.02,1.10)	210893	102.46	(9.22,242.79)	0.79	(0.76,0.82)
75 to 79	8435	6.40	(0.50,15.65)	0.94	(0.91,0.97)	164335	124.61	(11.00,291.48)	0.57	(0.54,0.60)
80 to 84	7415	8.47	(0.72,20.35)	0.90	(0.86,0.94)	114196	130.39	(12.37,303.67)	0.56	(0.53,0.59)
85 to 90	5605	12.26	(1.02,31.01)	1.20	(1.13,1.27)	65755	143.82	(13.16,350.98)	0.80	(0.75,0.85)
90 to 94	3354	18.75	(1.75,48.07)	1.61	(1.57,1.65)	32731	182.96	(18.28,460.70)	1.22	(1.18,1.25)
95 plus	1368	25.10	(2.27,64.37)	2.24	(2.15,2.32)	11173	205.00	(19.10,524.77)	2.12	(2.03,2.20)

Sheet S2

	Deaths					DALYs				
**Regions**	**CASE**	**ASMR**	**95%UI**	**AAPC**	**95%UI**	**CASE**	**ASMR**	**95%UI**	**AAPC**	**95%UI**

Andean Latin America	753	1.35	(0.06,3.36)	5.42	(5.30,5.52)	13735	24.06	(1.06,59.21)	4.80	(4.70,4.89)
Australasia	48	0.08	(0.01,0.19)	-0.53	(-0.69,-0.37)	1651	3.12	(0.48,6.40)	-0.88	(-0.96,-0.79)
Caribbean	844	1.56	(0.15,3.50)	1.86	(1.75,1.97)	18950	35.03	(4.07,75.62)	1.87	(1.78,1.95)
Central Asia	397	0.55	(0.21,0.90)	3.76	(3.51,3.98)	16129	20.22	(7.88,32.06)	1.75	(1.61,1.87)
Central Europe	666	0.28	(0.06,0.50)	-0.29	(-0.35,-0.23)	18867	8.19	(1.98,14.20)	-0.50	(-0.54,-0.47)
Central Latin America	4359	1.80	(0.26,3.51)	2.65	(2.47,2.78)	102991	41.33	(6.22,78.27)	2.60	(2.45,2.73)
Central Sub-Saharan Africa	295	0.71	(0.01,2.13)	-0.86	(-0.95,-0.77)	7822	16.03	(0.27,45.73)	-0.96	(-1.05,-0.87)
East Asia	6397	0.33	(0.00,0.98)	1.01	(0.96,1.06)	132666	6.21	(0.02,19.24)	1.17	(1.12,1.22)
Eastern Europe	764	0.21	(0.06,0.36)	2.74	(2.58,2.92)	26230	7.24	(1.88,12.04)	0.56	(0.48,0.63)
Eastern Sub-Saharan Africa	2129	1.59	(0.22,3.23)	2.04	(2.02,2.06)	49852	32.79	(4.93,66.16)	1.91	(1.89,1.93)
High-income Asia Pacific	4162	0.65	(0.15,1.17)	-2.11	(-2.23,-1.97)	73444	13.85	(3.62,24.20)	-2.07	(-2.16,-1.97)
High-income North America	3471	0.48	(0.01,1.50)	1.92	(1.73,2.06)	64433	9.32	(0.13,29.33)	0.92	(0.75,1.07)
North Africa and Middle East	3040	0.76	(0.04,1.96)	1.46	(1.42,1.52)	72473	16.65	(0.94,42.10)	1.35	(1.30,1.42)
Oceania	22	0.40	(0.00,1.42)	5.61	(5.56,5.65)	548	8.34	(0.03,30.71)	5.78	(5.71,5.83)
South Asia	11227	0.84	(0.09,1.76)	1.43	(1.35,1.51)	290428	20.13	(2.20,42.14)	1.28	(1.23,1.34)
Southeast Asia	3620	0.66	(0.01,1.91)	1.99	(1.96,2.02)	82204	13.76	(0.22,40.24)	1.84	(1.82,1.87)
Southern Latin America	580	0.63	(0.02,1.52)	2.25	(2.07,2.37)	10321	11.42	(0.34,27.88)	2.19	(2.04,2.31)

Southern Sub-Saharan Africa	265	0.55	(0.03,1.32)	2.02	(1.91,2.12)	7415	13.67	(0.97,31.68)	1.64	(1.55,1.72)
Tropical Latin America	2668	1.07	(0.09,2.32)	0.66	(0.56,0.80)	59901	23.40	(1.76,49.44)	0.43	(0.33,0.53)
Western Europe	4166	0.33	(0.05,0.65)	-0.39	(-0.46,-0.32)	78995	7.41	(1.17,13.74)	-1.07	(-1.10,-1.04)
Western Sub-Saharan Africa	817	0.53	(0.01,1.49)	3.38	(3.34,3.43)	22161	12.62	(0.26,33.57)	3.23	(3.20,3.26)

Sheet S3

	Deaths					DALYs				
**Countries**	**CASE**	**ASMR**	**95%UI**	**AAPC**	**95%UI**	**CASE**	**ASMR**	**95%UI**	**AAPC**	**95%UI**
American Samoa	1	2.02	(<0.01,8.78)	6.60	(6.48,6.71)	19	40.83	(<0.01,176.52)	5.89	(5.78,5.98)
Antigua and Barbuda	3	2.99	(0.22,6.95)	2.12	(1.85,2.35)	67	62.07	(5.79,142.21)	2.01	(1.75,2.23)
Arab Republic of Egypt	751	1.33	(0.02,3.84)	5.22	(5.04,5.37)	19050	30.06	(0.58,82.72)	5.43	(5.22,5.59)
Argentine Republic	355	0.61	(0.01,1.60)	2.74	(2.61,2.85)	6636	11.52	(0.13,29.47)	3.12	(2.99,3.25)
Australia	36	0.07	(0.01,0.17)	-0.60	(-0.88,-0.44)	1289	2.87	(0.41,6.07)	-0.87	(-0.98,-0.77)
Barbados	13	2.35	(0.15,5.47)	3.81	(3.62,3.98)	254	47.01	(3.79,104.69)	3.39	(3.21,3.54)
Belize	7	2.56	(0.16,6.25)	2.57	(2.37,2.74)	158	55.43	(3.30,130.80)	2.54	(2.37,2.69)
Bermuda	1	0.90	(0.04,2.10)	0.02	(-0.15,0.18)	27	18.78	(0.78,43.67)	-0.09	(-0.23,0.03)
Bolivarian Republic of	560	1.97	(0.30,4.07)	3.02	(2.78,3.27)	12440	41.87	(6.22,85.90)	2.07	(1.85,2.30)

Venezuela										
Bosnia and Herzegovina	41	0.62	(0.20,1.07)	0.49	(0.35,0.65)	1004	15.45	(5.53,25.55)	0.19	(0.08,0.31)
Brunei Darussalam	6	2.44	(0.23,5.54)	-1.43	(-1.55,-1.33)	138	46.39	(4.55,101.95)	-1.40	(-1.48,-1.31)
Burkina Faso	18	0.26	(<0.01,1.00)	1.43	(1.35,1.51)	410	5.24	(<0.01,20.03)	0.99	(0.93,1.05)
Canada	77	0.09	(<0.01,0.34)	-0.89	(-1.13,-0.72)	1557	2.03	(<0.01,6.93)	-1.59	(-1.77,-1.44)
Central African Republic	13	0.82	(0.01,2.48)	0.05	(<0.01,0.10)	371	19.04	(0.26,54.87)	-0.03	(-0.08,0.02)
Commonwealth of Dominica	3	3.14	(0.21,7.62)	1.86	(1.81,1.91)	58	67.22	(4.62,157.50)	1.87	(1.84,1.91)
Commonwealth of the Bahamas	9	2.34	(0.17,5.39)	1.97	(1.77,2.15)	211	51.54	(4.35,115.55)	1.86	(1.68,2.04)
Cook Islands	0	0.65	(<0.01,2.35)	6.21	(6.11,6.31)	3	12.86	(<0.01,46.08)	5.87	(5.78,5.95)
Czech Republic	46	0.19	(0.03,0.38)	-0.50	(-0.63,-0.35)	1333	5.93	(0.98,10.92)	-0.90	(-0.97,-0.81)
Democratic People's Republic of Korea	75	0.26	(<0.01,1.08)	0.22	(0.19,0.26)	1589	5.19	(<0.01,23.05)	-0.39	(-0.43,-0.35)
Democratic Republic of Sao Tome and Principe	1	1.10	(<0.01,3.41)	2.88	(2.81,2.93)	25	24.20	(0.01,69.39)	2.67	(2.63,2.72)
Democratic Republic of the Congo	171	0.60	(<0.01,2.03)	-1.54	(-1.67,-1.43)	4523	13.82	(<0.01,44.23)	-1.60	(-1.72,-1.49)
Democratic Republic of	4	0.51	(<0.01,2.08)	1.81	(1.74,1.87)	99	11.46	(<0.01,47.44)	1.66	(1.61,1.71)

Timor-Leste										
Democratic Socialist Republic of Sri Lanka	111	0.41	(<0.01,1.57)	2.00	(1.79,2.25)	2826	9.96	(<0.01,34.79)	2.85	(2.69,3.03)
Dominican Republic	117	1.21	(0.06,2.94)	2.24	(2.15,2.34)	2737	27.72	(1.83,65.32)	2.39	(2.27,2.52)
Eastern Republic of Uruguay	17	0.26	(<0.01,0.79)	0.52	(0.23,0.75)	305	5.12	(<0.01,14.88)	0.34	(0.08,0.54)
Federal Democratic Republic of Ethiopia	415	1.14	(0.04,3.15)	4.60	(4.56,4.63)	9004	22.98	(1.09,60.92)	4.55	(4.51,4.58)
Federal Democratic Republic of Nepal	85	0.37	(<0.01,1.07)	0.57	(0.52,0.61)	2504	10.46	(0.01,28.72)	0.23	(0.21,0.26)
Federal Republic of Germany	1769	0.67	(0.12,1.26)	1.21	(1.05,1.35)	29392	12.65	(2.40,23.26)	-0.21	(-0.35,-0.09)
Federal Republic of Nigeria	323	0.46	(0.01,1.30)	3.96	(3.85,4.07)	10122	12.35	(0.28,32.34)	3.91	(3.83,3.98)
Federal Republic of Somalia	89	2.03	(0.23,4.75)	2.76	(2.73,2.78)	2232	42.42	(5.10,94.95)	2.51	(2.49,2.54)
Federated States of Micronesia	0	0.31	(<0.01,2.20)	6.21	(6.06,6.35)	3	5.14	(<0.01,36.66)	6.75	(6.61,6.87)
Federative Republic of Brazil	2587	1.06	(0.09,2.31)	0.62	(0.52,0.75)	58102	23.21	(1.75,48.99)	0.40	(0.30,0.50)
French Republic	402	0.20	(0.02,0.43)	-0.36	(-0.45,-0.25)	7968	4.91	(0.59,10.07)	-0.49	(-0.56,-0.40)
Gabonese Republic	13	1.67	(0.01,4.89)	2.03	(1.97,2.10)	317	33.69	(0.26,94.60)	1.78	(1.71,1.84)
Georgia	41	0.65	(0.27,1.10)	4.81	(4.10,5.60)	1406	23.30	(9.94,37.77)	1.47	(1.13,1.78)

Grand Duchy of Luxembourg	6	0.47	(0.07,0.94)	0.45	(0.27,0.60)	122	10.87	(2.07,20.44)	0.02	(-0.06,0.10)
Greenland	0	0.16	(<0.01,0.57)	-1.95	(-2.04,-1.85)	2	3.61	(<0.01,12.78)	-1.95	(-2.03,-1.87)
Grenada	4	3.75	(0.28,8.79)	2.62	(2.45,2.76)	92	79.56	(5.72,179.00)	2.48	(2.36,2.58)
Guam	1	0.43	(<0.01,1.64)	4.59	(4.25,4.89)	22	10.09	(<0.01,41.15)	5.35	(5.12,5.56)
Hashemite Kingdom of Jordan	69	1.16	(0.01,3.23)	-0.35	(-0.51,-0.19)	1607	23.71	(0.40,61.99)	-0.27	(-0.46,-0.14)
Hellenic Republic	181	0.58	(0.05,1.27)	-2.33	(-2.77,-1.98)	3198	11.96	(1.16,24.61)	-1.75	(-1.99,-1.49)
Hungary	87	0.39	(0.10,0.71)	1.95	(1.78,2.12)	2082	9.89	(2.63,16.92)	0.59	(0.51,0.66)
Independent State of Papua New Guinea	2	0.05	(<0.01,0.26)	9.15	(9.07,9.26)	58	1.25	(<0.01,6.73)	9.70	(9.62,9.80)
Independent State of Samoa	1	0.72	(<0.01,3.32)	4.83	(4.76,4.89)	18	13.75	(<0.01,69.36)	5.30	(5.25,5.36)
Ireland	26	0.30	(0.02,0.61)	-0.64	(-0.89,-0.41)	686	8.12	(0.61,15.96)	-1.26	(-1.35,-1.16)
Islamic Republic of Afghanistan	72	0.95	(<0.01,3.06)	0.87	(0.85,0.90)	1829	22.00	(0.04,69.40)	0.77	(0.74,0.81)
Islamic Republic of Iran	164	0.24	(<0.01,0.75)	1.31	(1.20,1.41)	3910	5.40	(0.08,17.40)	1.14	(1.05,1.21)
Islamic Republic of Mauritania	8	0.46	(<0.01,1.64)	1.63	(1.56,1.69)	193	9.69	(<0.01,32.57)	1.56	(1.50,1.61)
Islamic Republic of Pakistan	1649	1.57	(0.20,3.20)	1.97	(1.94,2.00)	43548	37.24	(4.81,73.83)	2.02	(1.99,2.06)
Jamaica	50	1.62	(0.13,3.90)	3.56	(3.12,4.12)	1204	38.94	(4.37,90.81)	4.25	(3.77,4.77)
Japan	3967	0.76	(0.19,1.32)	-1.78	(-1.90,-1.64)	69651	17.00	(4.70,28.90)	-1.63	(-1.71,-1.53)

Kingdom of Bahrain	6	1.04	(0.01,2.87)	0.77	(0.65,0.89)	144	19.95	(0.38,55.92)	0.49	(0.41,0.57)
Kingdom of Belgium	84	0.28	(0.03,0.60)	0.78	(0.65,0.91)	2030	8.09	(1.00,15.89)	0.87	(0.79,0.99)
Kingdom of Bhutan	5	0.90	(0.06,2.16)	1.00	(0.96,1.04)	119	20.48	(1.21,48.67)	0.62	(0.58,0.65)
Kingdom of Cambodia	16	0.20	(<0.01,0.80)	0.25	(0.20,0.29)	305	3.33	(<0.01,14.39)	-0.47	(-0.50,-0.43)
Kingdom of Denmark	73	0.52	(0.10,0.96)	2.55	(2.34,2.78)	1494	11.33	(2.39,20.11)	0.92	(0.81,1.05)
Kingdom of Eswatini	4	0.80	(<0.01,2.50)	2.52	(2.44,2.60)	107	20.09	(0.22,57.60)	2.50	(2.45,2.55)
Kingdom of Lesotho	3	0.32	(<0.01,1.09)	3.68	(3.61,3.75)	88	8.47	(<0.01,26.66)	2.80	(2.75,2.85)
Kingdom of Morocco	488	1.58	(0.28,3.12)	1.87	(1.83,1.90)	10971	32.73	(6.57,64.34)	1.55	(1.52,1.58)
Kingdom of Norway	18	0.15	(0.02,0.31)	0.71	(0.45,1.07)	486	4.57	(0.60,8.81)	-0.94	(-1.02,-0.86)
Kingdom of Saudi Arabia	176	1.36	(<0.01,4.71)	1.77	(1.73,1.82)	4471	28.16	(<0.01,93.36)	1.23	(1.20,1.28)
Kingdom of Spain	394	0.29	(0.03,0.64)	-2.55	(-2.66,-2.44)	7524	6.80	(0.86,13.28)	-2.20	(-2.30,-2.11)
Kingdom of Sweden	107	0.37	(0.06,0.74)	2.10	(1.90,2.25)	1829	6.94	(1.10,12.83)	0.22	(0.09,0.35)
Kingdom of Thailand	132	0.12	(<0.01,0.76)	2.59	(2.40,2.81)	2750	2.51	(<0.01,15.17)	2.11	(1.89,2.33)
Kingdom of the Netherlands	97	0.24	(0.01,0.55)	2.34	(2.16,2.61)	2277	5.99	(0.63,12.81)	1.34	(1.24,1.47)
Kingdom of Tonga	1	0.81	(<0.01,3.29)	6.45	(6.32,6.57)	13	17.13	(<0.01,71.08)	6.15	(6.02,6.26)
Kyrgyz Republic	14	0.34	(0.12,0.60)	4.17	(3.83,4.56)	617	13.66	(4.99,23.39)	1.30	(1.14,1.46)
Lao People's Democratic	19	0.53	(<0.01,2.29)	-0.17	(-0.26,-0.09)	421	10.62	(<0.01,47.24)	0.25	(0.17,0.34)

Republic										
Lebanese Republic	21	0.35	(<0.01,1.25)	-0.81	(-0.94,-0.68)	520	9.02	(0.01,28.13)	0.25	(0.17,0.34)
Malaysia	340	1.33	(<0.01,3.94)	4.17	(3.83,4.56)	7845	28.52	(<0.01,87.04)	-0.93	(-1.00,-0.88)
Mongolia	11	0.58	(0.20,1.01)	-0.17	(-0.26,-0.09)	481	21.15	(7.64,34.59)	-0.67	(-0.73,-0.60)
Montenegro	3	0.30	(0.01,0.78)	-0.81	(-0.94,-0.68)	76	7.56	(0.33,17.69)	0.11	(0.05,0.16)
New Zealand	12	0.14	(0.02,0.31)	4.17	(3.83,4.56)	362	4.39	(0.61,8.95)	-0.97	(-1.22,-0.76)
North Macedonia	10	0.33	(0.04,0.69)	-0.17	(-0.26,-0.09)	353	10.43	(1.53,20.25)	0.03	(-0.01,0.07)
Northern Mariana Islands	1	1.70	(<0.01,7.10)	-0.81	(-0.94,-0.68)	15	32.43	(<0.01,127.70)	6.44	(6.33,6.52)
Palestine	8	0.35	(<0.01,1.41)	4.17	(3.83,4.56)	185	7.74	(<0.01,28.84)	-0.76	(-0.83,-0.70)
People's Democratic Republic of Algeria	187	0.68	(<0.01,2.07)	-0.17	(-0.26,-0.09)	3604	11.53	(<0.01,33.21)	0.01	(-0.04,0.07)
People's Republic of Bangladesh	780	0.62	(0.06,1.29)	-0.81	(-0.94,-0.68)	19908	14.74	(1.29,30.68)	1.71	(1.54,1.88)
People's Republic of China	6232	0.33	(<0.01,0.99)	4.17	(3.83,4.56)	129341	6.28	(0.02,19.56)	1.22	(1.17,1.27)
Plurinational State of Bolivia	149	1.92	(0.05,5.36)	-0.17	(-0.26,-0.09)	2926	34.54	(1.32,91.64)	2.95	(2.89,3.01)
Portuguese Republic	119	0.38	(0.02,0.84)	-0.81	(-0.94,-0.68)	2165	7.94	(0.62,16.36)	-1.37	(-1.50,-1.26)
Principality of Andorra	1	0.34	(0.04,0.72)	-1.40	(-1.56,-1.24)	13	8.32	(1.09,16.04)	-1.14	(-1.24,-1.04)
Principality of Monaco	0	0.33	(0.05,0.70)	0.85	(0.82,0.89)	9	8.03	(1.20,15.66)	0.15	(0.13,0.18)

Puerto Rico	197	2.44	(0.16,5.48)	0.57	(0.34,0.78)	3731	51.33	(3.47,114.26)	0.77	(0.54,0.97)
Republic of Albania	15	0.35	(0.10,0.63)	-0.32	(-0.42,-0.22)	443	9.88	(3.24,16.75)	-0.30	(-0.35,-0.25)
Republic of Angola	65	0.75	(0.02,2.14)	0.68	(0.63,0.72)	1787	17.24	(0.39,48.39)	0.46	(0.42,0.50)
Republic of Armenia	21	0.47	(0.16,0.80)	8.85	(8.08,9.43)	761	17.15	(6.49,28.25)	2.55	(2.25,2.79)
Republic of Austria	119	0.52	(0.04,1.07)	2.19	(2.05,2.33)	2164	10.64	(1.27,20.88)	0.89	(0.81,0.97)
Republic of Azerbaijan	42	0.48	(0.16,0.83)	2.69	(2.60,2.76)	1871	18.97	(7.11,31.11)	1.46	(1.41,1.51)
Republic of Belarus	12	0.07	(0.02,0.14)	4.12	(3.68,4.45)	1027	6.23	(1.55,10.67)	0.77	(0.67,0.84)
Republic of Benin	13	0.33	(<0.01,1.20)	2.01	(1.93,2.08)	331	7.29	(<0.01,26.68)	1.88	(1.81,1.95)
Republic of Botswana	5	0.38	(<0.01,1.17)	1.76	(1.63,1.88)	147	10.56	(<0.01,30.35)	1.31	(1.22,1.40)
Republic of Bulgaria	73	0.47	(0.10,0.89)	2.58	(2.30,2.93)	1930	13.15	(3.19,23.59)	1.74	(1.57,2.00)
Republic of Burundi	64	1.75	(0.16,3.74)	1.58	(1.56,1.61)	1511	35.45	(3.69,74.47)	1.29	(1.25,1.32)
Republic of Cabo Verde	2	0.49	(<0.01,1.57)	4.31	(4.16,4.44)	46	10.90	(<0.01,33.19)	3.42	(3.30,3.54)
Republic of Cameroon	132	1.31	(<0.01,3.70)	10.49	(10.30,10.66)	3443	30.04	(0.05,80.65)	10.34	(10.13,10.53)
Republic of Chad	8	0.17	(<0.01,0.79)	1.02	(0.98,1.06)	205	4.00	(<0.01,17.12)	0.83	(0.80,0.87)
Republic of Chile	208	0.78	(0.03,1.67)	1.11	(0.95,1.24)	3380	12.77	(0.50,28.04)	0.46	(0.31,0.59)
Republic of Colombia	445	0.81	(0.08,1.61)	0.39	(0.17,0.60)	10180	18.58	(2.06,36.97)	0.54	(0.37,0.67)
Republic of Costa Rica	87	1.59	(0.17,3.15)	2.01	(1.76,2.37)	2023	36.59	(4.57,71.82)	1.60	(1.39,1.86)

Republic of Croatia	42	0.41	(0.06,0.81)	0.52	(0.27,0.80)	962	10.10	(2.01,18.85)	0.12	(-0.10,0.27)
Republic of Cuba	132	0.65	(0.01,1.77)	2.66	(2.50,2.83)	2698	13.56	(0.27,36.50)	1.91	(1.75,2.06)
Republic of Cyprus	13	0.72	(0.09,1.53)	-2.45	(-2.59,-2.32)	271	13.58	(2.13,25.80)	-2.01	(-2.12,-1.90)
Republic of Cote d’Ivoire	35	0.41	(<0.01,1.35)	2.10	(2.04,2.16)	1012	9.91	(<0.01,31.92)	1.93	(1.87,1.99)
Republic of Djibouti	11	2.41	(0.22,5.17)	3.55	(3.53,3.58)	256	47.50	(5.56,99.41)	3.23	(3.20,3.25)
Republic of Ecuador	162	1.08	(0.03,3.16)	4.53	(4.32,4.75)	3106	19.63	(0.39,55.39)	4.28	(4.06,4.54)
Republic of El Salvador	165	2.65	(0.25,5.62)	4.22	(4.04,4.38)	3584	59.05	(5.75,126.30)	3.95	(3.79,4.08)
Republic of Equatorial Guinea	6	1.46	(0.04,3.98)	2.11	(2.00,2.21)	141	31.53	(1.18,82.05)	1.72	(1.60,1.82)
Republic of Estonia	22	0.65	(0.13,1.21)	4.83	(4.41,5.23)	480	15.98	(4.01,27.91)	2.30	(2.13,2.46)
Republic of Fiji	12	1.70	(<0.01,6.13)	7.83	(7.75,7.91)	290	37.48	(<0.01,131.73)	7.53	(7.46,7.59)
Republic of Finland	55	0.33	(0.10,0.58)	0.23	(0.07,0.37)	1161	8.06	(2.29,13.34)	-0.77	(-0.87,-0.69)
Republic of Ghana	158	1.21	(0.01,3.16)	3.18	(3.11,3.25)	3567	24.20	(0.37,61.88)	2.66	(2.60,2.72)
Republic of Guatemala	182	1.68	(0.16,3.64)	4.36	(4.14,4.63)	4897	44.10	(4.73,92.80)	4.72	(4.55,4.90)
Republic of Guinea	5	0.09	(<0.01,0.42)	2.64	(2.56,2.73)	157	2.78	(<0.01,11.52)	2.90	(2.81,2.99)
Republic of Guinea-Bissau	2	0.45	(<0.01,1.52)	1.83	(1.79,1.88)	65	10.28	(<0.01,34.36)	1.62	(1.59,1.66)
Republic of Guyana	21	3.44	(0.33,8.13)	2.63	(2.40,2.81)	522	78.85	(8.17,182.25)	2.80	(2.57,2.99)
Republic of Haiti	160	2.40	(0.27,6.57)	1.76	(1.71,1.81)	4335	59.27	(7.93,154.39)	1.75	(1.71,1.81)

Republic of Honduras	57	0.98	(0.09,2.10)	3.25	(3.12,3.38)	1507	24.29	(2.37,50.37)	2.51	(2.40,2.63)
Republic of Iceland	1	0.08	(<0.01,0.19)	1.66	(1.50,1.85)	17	2.82	(0.18,6.25)	0.97	(0.87,1.07)
Republic of India	8708	0.80	(0.08,1.72)	1.49	(1.41,1.57)	224348	19.26	(2.02,41.42)	1.23	(1.15,1.30)
Republic of Indonesia	1951	1.16	(0.05,2.84)	2.10	(2.06,2.13)	46244	23.37	(0.74,59.40)	1.80	(1.77,1.82)
Republic of Iraq	307	1.51	(0.02,4.19)	0.94	(0.88,1.00)	7640	33.39	(0.61,88.73)	0.57	(0.52,0.63)
Republic of Italy	262	0.13	(0.01,0.34)	-3.23	(-3.35,-3.10)	5482	3.38	(0.27,7.64)	-3.55	(-3.64,-3.46)
Republic of Kazakhstan	114	0.73	(0.30,1.22)	3.91	(3.53,4.26)	4239	24.48	(9.86,39.75)	1.55	(1.39,1.71)
Republic of Kenya	394	2.29	(0.45,4.34)	2.08	(2.05,2.11)	8924	44.29	(8.48,81.98)	1.92	(1.89,1.94)
Republic of Kiribati	0	0.45	(<0.01,2.30)	4.64	(4.57,4.71)	3	6.90	(<0.01,39.55)	3.78	(3.72,3.83)
Republic of Korea	153	0.16	(<0.01,0.60)	-4.06	(-4.18,-3.94)	2954	3.06	(<0.01,10.79)	-4.38	(-4.53,-4.21)
Republic of Latvia	16	0.33	(0.10,0.59)	5.11	(4.87,5.31)	483	11.33	(3.53,18.62)	1.68	(1.61,1.75)
Republic of Liberia	8	0.50	(<0.01,1.93)	1.72	(1.64,1.83)	200	10.99	(<0.01,39.80)	1.56	(1.47,1.64)
Republic of Lithuania	13	0.20	(0.05,0.35)	4.67	(4.34,4.93)	528	8.80	(2.29,14.60)	1.23	(1.15,1.30)
Republic of Madagascar	105	1.30	(0.13,2.89)	2.65	(2.61,2.69)	2635	27.09	(3.19,57.65)	2.35	(2.31,2.39)
Republic of Malawi	162	2.74	(0.36,5.55)	2.02	(1.99,2.06)	3790	56.44	(8.60,107.30)	1.50	(1.47,1.54)
Republic of Maldives	1	0.56	(<0.01,2.06)	-0.52	(-0.62,-0.41)	35	12.18	(<0.01,45.64)	-1.00	(-1.10,-0.89)
Republic of Mali	20	0.35	(<0.01,1.23)	1.62	(1.57,1.68)	441	6.48	(<0.01,23.20)	1.36	(1.32,1.40)

Republic of Malta	5	0.43	(0.05,0.87)	-0.68	(-0.82,-0.51)	110	10.43	(1.57,19.97)	-0.87	(-0.98,-0.77)
Republic of Mauritius	32	1.77	(<0.01,6.00)	7.69	(7.44,7.96)	669	35.26	(<0.01,121.82)	7.47	(7.22,7.74)
Republic of Moldova	7	0.12	(0.04,0.21)	3.10	(2.68,3.52)	664	10.84	(3.62,18.58)	1.49	(1.42,1.56)
Republic of Mozambique	174	2.01	(0.26,4.39)	2.87	(2.83,2.90)	4454	43.01	(6.66,90.42)	2.33	(2.30,2.36)
Republic of Namibia	3	0.24	(<0.01,0.80)	0.81	(0.75,0.88)	86	6.61	(<0.01,20.48)	0.50	(0.44,0.55)
Republic of Nauru	0	2.04	(<0.01,7.78)	5.29	(5.26,5.32)	3	46.76	(<0.01,164.52)	5.50	(5.46,5.53)
Republic of Nicaragua	106	2.30	(0.23,4.95)	2.72	(2.55,2.86)	2819	57.81	(5.95,122.52)	2.53	(2.39,2.65)
Republic of Niue	0	1.38	(<0.01,7.22)	3.55	(3.51,3.60)	1	25.09	(<0.01,128.81)	3.47	(3.43,3.53)
Republic of Palau	0	1.35	(<0.01,5.68)	6.95	(6.86,7.03)	5	24.89	(<0.01,111.56)	6.54	(6.47,6.59)
Republic of Panama	63	1.41	(0.20,2.97)	3.53	(3.28,3.78)	1407	32.01	(4.24,62.74)	2.80	(2.59,3.00)
Republic of Paraguay	81	1.51	(0.13,3.52)	2.08	(1.95,2.21)	1799	31.87	(3.19,71.85)	1.90	(1.79,2.00)
Republic of Peru	442	1.35	(0.03,3.51)	6.89	(6.64,7.09)	7703	23.68	(0.62,59.92)	6.03	(5.84,6.18)
Republic of Poland	120	0.16	(0.02,0.31)	-3.01	(-3.19,-2.85)	3775	5.13	(0.76,9.68)	-2.51	(-2.61,-2.43)
Republic of Rwanda	61	1.31	(0.11,3.31)	0.91	(0.88,0.94)	1382	25.25	(2.41,61.65)	0.40	(0.36,0.44)
Republic of San Marino	0	0.14	(0.02,0.30)	-1.85	(-2.30,-1.60)	4	5.18	(0.74,10.17)	-1.05	(-1.24,-0.95)
Republic of Senegal	38	0.62	(<0.01,2.11)	0.43	(0.32,0.53)	782	11.69	(<0.01,38.27)	-0.16	(-0.28,-0.05)
Republic of Serbia	80	0.44	(0.01,1.03)	-0.71	(-0.86,-0.59)	1818	10.39	(0.23,23.34)	-0.67	(-0.79,-0.57)

Republic of Seychelles	1	1.02	(<0.01,3.95)	1.89	(1.55,2.21)	22	20.06	(<0.01,75.97)	1.36	(1.11,1.59)
Republic of Sierra Leone	16	0.52	(<0.01,1.59)	-0.24	(-0.27,-0.22)	418	12.17	(0.05,35.50)	-0.05	(-0.06,-0.04)
Republic of Singapore	36	0.44	(0.03,1.16)	-3.76	(-3.98,-3.55)	701	8.32	(0.60,21.37)	-4.14	(-4.36,-3.93)
Republic of Slovenia	10	0.19	(0.05,0.37)	-0.35	(-0.65,-0.06)	293	6.23	(1.51,10.71)	-0.64	(-0.79,-0.50)
Republic of South Africa	211	0.54	(0.03,1.29)	1.63	(1.46,1.78)	5814	13.26	(0.90,30.33)	1.16	(1.01,1.29)
Republic of South Sudan	74	2.51	(0.28,5.87)	3.65	(3.60,3.70)	1749	51.63	(6.07,117.61)	3.46	(3.41,3.50)
Republic of Sudan	46	0.27	(<0.01,1.04)	2.20	(2.13,2.25)	1174	6.46	(<0.01,24.39)	1.75	(1.67,1.81)
Republic of Suriname	9	1.49	(0.06,4.04)	0.97	(0.81,1.12)	205	32.18	(1.48,84.76)	0.91	(0.77,1.05)
Republic of Tajikistan	4	0.07	(0.02,0.15)	1.85	(1.76,1.95)	443	8.24	(2.10,15.33)	0.89	(0.85,0.94)
Republic of the Congo	26	1.27	(0.02,3.59)	0.25	(0.19,0.30)	683	28.15	(0.44,75.94)	0.15	(0.10,0.20)
Republic of the Gambia	4	0.47	(<0.01,1.58)	3.58	(3.44,3.74)	96	10.70	(<0.01,33.67)	3.48	(3.32,3.65)
Republic of the Marshall Islands	0	0.69	(<0.01,3.59)	5.52	(5.49,5.55)	3	11.93	(<0.01,71.39)	4.96	(4.91,5.01)
Republic of the Niger	11	0.17	(<0.01,0.59)	1.45	(1.41,1.49)	316	4.30	(<0.01,14.62)	1.44	(1.41,1.48)
Republic of the Philippines	278	0.41	(<0.01,1.41)	0.71	(0.63,0.83)	6132	8.31	(0.05,29.49)	0.80	(0.74,0.86)
Republic of the Union of Myanmar	199	0.50	(<0.01,1.65)	0.71	(0.68,0.74)	4364	10.05	(<0.01,35.34)	0.47	(0.44,0.50)
Republic of Trinidad and	76	3.84	(0.61,7.84)	2.08	(1.88,2.26)	1712	85.06	(12.49,170.90)	2.35	(2.16,2.52)

Tobago										
Republic of Tunisia	91	0.73	(0.06,1.93)	1.02	(0.96,1.08)	2127	15.95	(1.60,40.94)	0.80	(0.74,0.85)
Republic of Turkey	425	0.47	(<0.01,1.46)	-0.08	(-0.38,0.16)	9463	10.03	(<0.01,30.52)	-0.29	(-0.59,-0.04)
Republic of Uganda	228	2.00	(0.20,4.57)	0.24	(0.19,0.29)	5079	39.69	(4.48,85.27)	0.12	(0.07,0.17)
Republic of Uzbekistan	122	0.52	(0.17,0.90)	4.44	(3.60,5.17)	5233	19.80	(6.88,32.91)	2.77	(2.51,3.06)
Republic of Vanuatu	2	1.96	(<0.01,5.51)	2.56	(2.53,2.60)	55	37.77	(<0.01,113.32)	2.46	(2.43,2.49)
Republic of Yemen	34	0.29	(<0.01,0.86)	0.41	(0.38,0.43)	891	6.77	(0.07,19.00)	0.41	(0.38,0.43)
Republic of Zambia	94	1.61	(0.10,3.97)	2.84	(2.78,2.89)	2397	36.73	(3.12,86.45)	3.01	(2.96,3.06)
Republic of Zimbabwe	40	0.76	(0.01,2.07)	4.24	(4.13,4.38)	1172	18.77	(0.47,47.42)	3.91	(3.81,4.02)
Romania	96	0.23	(0.08,0.37)	1.77	(1.47,2.01)	3596	9.36	(3.37,14.78)	0.63	(0.44,0.78)
Russian Federation	652	0.27	(0.07,0.46)	1.97	(1.68,2.20)	18451	7.50	(2.01,12.45)	0.33	(0.21,0.44)
Saint Kitts and Nevis	3	4.04	(0.31,9.63)	1.53	(1.37,1.71)	63	87.69	(7.96,196.03)	1.42	(1.28,1.59)
Saint Lucia	6	2.41	(0.15,5.61)	-0.16	(-0.26,-0.05)	123	50.56	(3.21,115.29)	-0.07	(-0.18,0.05)
Saint Vincent and the Grenadines	3	2.04	(0.14,4.76)	1.83	(1.69,1.95)	66	44.64	(3.30,100.26)	1.86	(1.70,2.00)
Slovak Republic	33	0.33	(0.07,0.65)	-0.94	(-1.05,-0.86)	927	9.44	(1.93,17.20)	-0.93	(-1.00,-0.88)
Socialist Republic of Viet Nam	530	0.66	(<0.01,2.28)	0.69	(0.58,0.80)	10377	12.08	(<0.01,42.10)	4.22	(4.12,4.32)

Solomon Islands	0	0.22	(<0.01,0.99)	-0.94	(-1.05,-0.86)	9	3.83	(<0.01,18.31)	3.66	(3.49,3.81)
State of Eritrea	30	1.72	(0.15,3.99)	0.69	(0.58,0.80)	689	32.09	(3.20,75.87)	2.00	(1.98,2.03)
State of Israel	115	0.82	(0.06,1.79)	-0.94	(-1.05,-0.86)	2050	15.49	(1.27,31.53)	-0.64	(-0.90,-0.34)
State of Kuwait	6	0.29	(<0.01,0.95)	0.69	(0.58,0.80)	122	5.39	(<0.01,16.64)	-3.50	(-3.81,-3.10)
State of Libya	54	1.17	(0.04,3.12)	-0.94	(-1.05,-0.86)	1397	27.21	(1.19,71.03)	2.34	(2.26,2.40)
State of Qatar	4	0.91	(<0.01,2.60)	0.69	(0.58,0.80)	112	17.98	(<0.01,49.52)	0.44	(0.18,0.69)
Sultanate of Oman	16	1.01	(0.01,2.81)	-0.94	(-1.05,-0.86)	419	22.95	(0.19,60.73)	7.96	(7.80,8.11)
Swiss Confederation	30	0.12	(<0.01,0.38)	0.69	(0.58,0.80)	613	2.96	(0.04,8.35)	-0.67	(-0.73,-0.60)
Syrian Arab Republic	99	0.83	(0.01,2.36)	0.86	(0.78,0.94)	2404	18.12	(0.35,52.06)	0.69	(0.62,0.76)
Taiwan (Province of China)	90	0.21	(<0.01,1.16)	-1.18	(-1.77,-0.72)	1736	4.01	(<0.01,20.86)	-0.97	(-1.49,-0.54)
Togolese Republic	13	0.49	(<0.01,1.56)	1.26	(1.22,1.30)	331	10.57	(<0.01,32.59)	0.98	(0.94,1.02)
Tokelau	0	0.88	(<0.01,3.75)	7.07	(6.91,7.22)	0	17.30	(<0.01,74.20)	6.94	(6.85,7.02)
Turkmenistan	29	0.79	(0.31,1.38)	3.04	(2.81,3.28)	1077	27.08	(11.00,43.84)	1.84	(1.70,1.97)
Tuvalu	0	0.87	(<0.01,3.67)	7.23	(7.11,7.34)	2	17.20	(<0.01,68.62)	6.96	(6.82,7.08)
Ukraine	41	0.05	(0.01,0.10)	9.89	(9.25,10.50)	4598	5.75	(1.16,10.51)	0.78	(0.73,0.83)
Union of the Comoros	8	2.00	(0.22,4.57)	1.85	(1.81,1.89)	180	39.98	(4.74,86.86)	1.45	(1.39,1.51)
United Arab Emirates	13	0.85	(<0.01,2.83)	0.54	(0.03,0.98)	365	16.94	(<0.01,54.16)	-0.14	(-0.48,0.15)

United Kingdom of Great Britain and Northern Ireland	284	0.18	(0.03,0.33)	-1.51	(-1.67,-1.27)	7858	5.64	(0.98,10.57)	-2.05	(-2.11,-1.99)
United Mexican States	2694	2.22	(0.33,4.40)	2.89	(2.68,3.07)	64133	50.80	(8.19,101.00)	2.98	(2.73,3.16)
United Republic of Tanzania	218	1.01	(0.22,1.94)	1.36	(1.32,1.40)	5528	22.89	(5.91,43.65)	1.41	(1.38,1.43)
United States of America	3394	0.53	(0.01,1.64)	2.07	(1.87,2.22)	62872	10.23	(0.14,32.29)	1.05	(0.87,1.20)
United States Virgin Islands	2	1.08	(0.07,2.69)	0.33	(0.14,0.49)	46	24.54	(2.08,56.39)	0.50	(0.33,0.65)
